# Interference-aware optimization of three-tier RIS-enhanced hierarchical aerial computing: integrating terrestrial base stations for persistent 6G IoT coverage

**DOI:** 10.1038/s41598-026-62437-y

**Published:** 2026-07-22

**Authors:** Basma Diaa, Ibrahim I. Ibrahim, Ahmed M. Abd El-Haleem, Mostafa M. Abdelhakam

**Affiliations:** Department of Electronics and Communications Engineering, Faculty of Engineering, Capital University (Formerly Helwan University), Cairo, Egypt

**Keywords:** Aerial computing, Unmanned aerial vehicle (UAV), High altitude platform (HAP), Reconfigurable intelligent surfaces (RIS), Base station (BS), Mobile edge computing (MEC), Resource allocation, Matching game theory, Riemannian conjugate gradient (RCG), 6G, Internet of Things, Co-channel interference (CCI), Engineering, Mathematics and computing

## Abstract

The proliferation of Internet of Things (IoT) devices in emerging 6G networks demands computing architectures that simultaneously deliver high throughput, low latency, and persistent coverage across heterogeneous deployment environments. Existing two-tier unmanned aerial vehicle–high-altitude platform (UAV–HAP) frameworks offer flexible edge processing but suffer from limited battery endurance, constrained computational capacity, and susceptibility to co-channel interference (CCI) when multiple aerial platforms share the same spectrum. This paper proposes a novel three-tier RIS-enhanced hierarchical aerial computing architecture that integrates a grid-powered reconfigurable intelligent surface–equipped base station (BS-RIS) alongside four RIS-equipped UAVs and a stratospheric HAP, so as to provide persistent, interference-managed 6G IoT coverage. The proposed architecture introduces a sub-array RIS partitioning mechanism in which each RIS panel, consisting of 256 elements divided into 4 sub-arrays, dedicates one sub-array per neighboring interfering platform, achieving 85 % inter-platform interference suppression (residual fraction $\psi ^{\textrm{sup}}=0.15$). A comprehensive signal-to-interference-plus-noise ratio model is derived that captures both intra-platform CCI and inter-platform interference across all tiers. The resulting joint mixed-integer nonlinear programming problem is decomposed into three sequential stages: (i) a three-way hotspot-aware stable matching algorithm that associates IoT devices to platforms while penalising interference-heavy assignments; (ii) a sub-array-aware Riemannian conjugate gradient phase optimization that simultaneously enhances desired signal gains and suppresses inter-platform leakage; and (iii) a platform-aware hierarchical task distribution algorithm applying differentiated local-processing thresholds for battery-constrained UAVs (70 % delay margin) versus the grid-powered BS (100 % threshold). Extensive Monte Carlo simulations demonstrate that the proposed framework achieves approximately $30\,\%$ higher total computed data volume, $15\,\%$ points higher task completion rate, and $20\,\%$ lower average end-to-end delay compared to the two-tier UAV–HAP.

## Introduction

The global proliferation of Internet of Things (IoT) devices, projected to exceed 100 billion connected nodes by 2030 under 6G deployment scenarios, imposes unprecedented demands on wireless infrastructure in terms of ultra-low latency, massive connectivity, and energy efficiency^1,2^. Conventional terrestrial cellular networks, while offering high computational capacity and persistent coverage, are fundamentally ill-suited to serve dynamic, geographically dispersed IoT deployments in precision agriculture, industrial automation, disaster response, and remote environmental monitoring, where fixed base-station coverage is either unavailable or prohibitively expensive to extend^3^. Unmanned aerial vehicles (UAVs) equipped with mobile edge computing (MEC) servers have emerged as a compelling solution for on-demand, proximity-driven IoT offloading, owing to their mobility, line-of-sight channel advantages, and rapid deployment capabilities^4^. However, UAV-based architectures are fundamentally constrained by limited battery endurance (30–90 min of flight time), restricted onboard computational capacity, and susceptibility to inter-platform CCI when multiple UAVs are co-deployed over the same coverage area. High-altitude platform stations (HAP), operating at stratospheric altitudes of 18 km, offer wide-area coverage and cloud-scale computational resources but are unsuitable as direct IoT access points due to the long propagation distances and associated high path loss^5^. Reconfigurable intelligent surfaces (RIS) have attracted substantial research attention as a passive, low-cost means of enhancing wireless channel quality through programmable electromagnetic reflection^6^. By configuring the phase shifts of large arrays of reflecting elements, RIS panels can coherently combine multipath components to strengthen desired signal paths and introduce destructive interference along undesired interference directions^7^. The integration of RIS with aerial platforms like UAVs has also demonstrated considerable improvement in terms of communication range, energy efficiency, and spectral efficiency^8^. However, the existing RIS-UAV framework only focuses on signal enhancement, whereas the important issue of inter-platform CCI management in the context of shared spectrum among multiple UAVs is still untouched.

### Related work

A STAR-RIS and UAV combination for simultaneous task offloading and communications (STOC) in MEC networks was proposed in^9^, jointly optimizing beamforming, time scheduling, and UAV trajectory. However, the single-UAV single-BS configuration does not address inter-platform CCI in multi-UAV shared-spectrum deployments, nor the HAP cloud tier for overflow offloading that characterises our three-tier architecture. A priority-aware layered offloading framework for UAV-assisted MEC was proposed in^10^, deriving closed-form water-filling solutions under TDMA-based prioritisation. While important reliability-latency trade-offs were established, a single-UAV architecture without RIS enhancement or multi-platform interference management was assumed. The integration of RIS with non-terrestrial networks (NTNs) for high-mobility UAV scenarios was investigated in^11^, where up to 15 dB SINR gains were demonstrated with increasing RIS elements but at quadratic computational cost. This finding validates our sub-array partitioning design, which distributes optimization complexity across dedicated sub-arrays. Through field measurements in^12^, it was demonstrated that active RIS achieves 40% higher data rates than passive configurations but consumes more power, directly justifying the adoption of passive RIS for battery-constrained UAV platforms to maximize flight endurance and service continuity.

A UAV-mounted RIS hierarchical framework with energy-aware trajectory optimization using RCG methods was studied in^2^, where 95% task completion were achieved. Nevertheless, that framework remained constrained to UAV–HAP architectures without terrestrial BS integration, limiting persistent coverage and introducing service gaps during UAV battery replenishment. Next-generation UAV communications for 6G were surveyed in^3^, where hybrid terrestrial-aerial architectures and RIS-equipped base stations were explicitly advocated to complement UAV platforms, directly motivating our three-tier framework. Cooperative multi-UAV edge computing with RIS assistance was investigated in^13^, achieving 25% performance gains. However, the distributed consensus algorithm exhibits $\mathscr {O}(|\mathscr {U}|^3)$ complexity, whereas the stable matching framework proposed here maintains polynomial $\mathscr {O}(|\mathscr {I}|^2(|\mathscr {U}|+1))$ complexity scalable to hundreds of IoT devices. Secrecy rates in IRS-assisted UAV communications were maximized in^14^, focusing exclusively on physical layer security rather than computation offloading, with unlimited UAV energy budgets assumed. A Stackelberg game-theoretic approach to dynamic computation offloading was developed in^15^, achieving 15% energy savings, but homogeneous edge servers were assumed, failing to capture the fundamental UAV–BS energy asymmetry.

The above review reveals that existing works on RIS-assisted aerial computing either focus on single-tier UAV–HAP architectures without terrestrial infrastructure, or address signal enhancement without explicit inter-platform CCI management under shared-spectrum operation. Specifically, no prior work simultaneously addresses: (i) persistent coverage through grid-powered terrestrial BS integration eliminating service interruptions caused by UAV battery constraints; (ii) inter-platform CCI suppression via dedicated RIS sub-array partitioning under shared-spectrum multi-UAV deployment; and (iii) heterogeneous task distribution reflecting the asymmetric energy profiles of battery-constrained UAVs versus grid-powered BS. This paper fills this gap by proposing a three-tier BS-RIS–UAV-RIS–HAP architecture with a three-stage optimization framework that jointly solves association, RIS phase configuration, and task allocation under explicit interference management.

### Contributions

The main contributions of this paper are as follows:

1. A novel three-tier hierarchical aerial computing architecture is proposed, consisting of RIS-equipped UAVs, a grid-powered BS-RIS, and a HAP at 18 km altitude providing cloud computing over orthogonal backhaul links.
2. We introduce a sub-array RIS architecture in which each RIS panel ($N = 256$ elements) is partitioned into $M = 4$ equal sub-arrays, each dedicated to suppressing inter-platform interference from one neighbouring platform through destructive phase alignment. This mechanism achieves 85% interference suppression ($\psi ^{\textrm{sup}} = 0.15$) while preserving signal enhancement capability.
3. A comprehensive SINR model is derived, incorporating both intra-platform CCI and inter-platform interference terms for both UAV-associated and BS-associated devices, together with a sub-array suppression factor $\psi ^{\textrm{sup}} \in [0,1]$ that couples the physical-layer RIS design to the system-level interference model.
4. A three-stage sequential decomposition is proposed, comprising: (i) a three-way hotspot-aware stable matching algorithm with inter-platform interference penalties and spatial proximity bonuses; (ii) a sub-array-aware RCG algorithm with extended gradient computation capturing inter-platform interference sensitivity; and (iii) a platform-aware task distribution algorithm with differentiated thresholds for battery-constrained UAVs versus grid-powered BS.
5. Extensive simulations demonstrate approximately $30\,\%$ higher computed data volume, 15% higher task completion rate, and $20\,\%$ lower average delay versus the two-tier architecture, with 8–12 dB SINR improvement over unmanaged interference scenarios.

The rest of this paper is organised as follows. Section "System model" presents the system model, channel model, and problem formulation. Section "Proposed algorithms" describes the three proposed algorithms Section "Results and discussion" presents simulation results. Section Conclusion concludes the paper and outlines directions for future research.

## System model

**Fig. 1:**
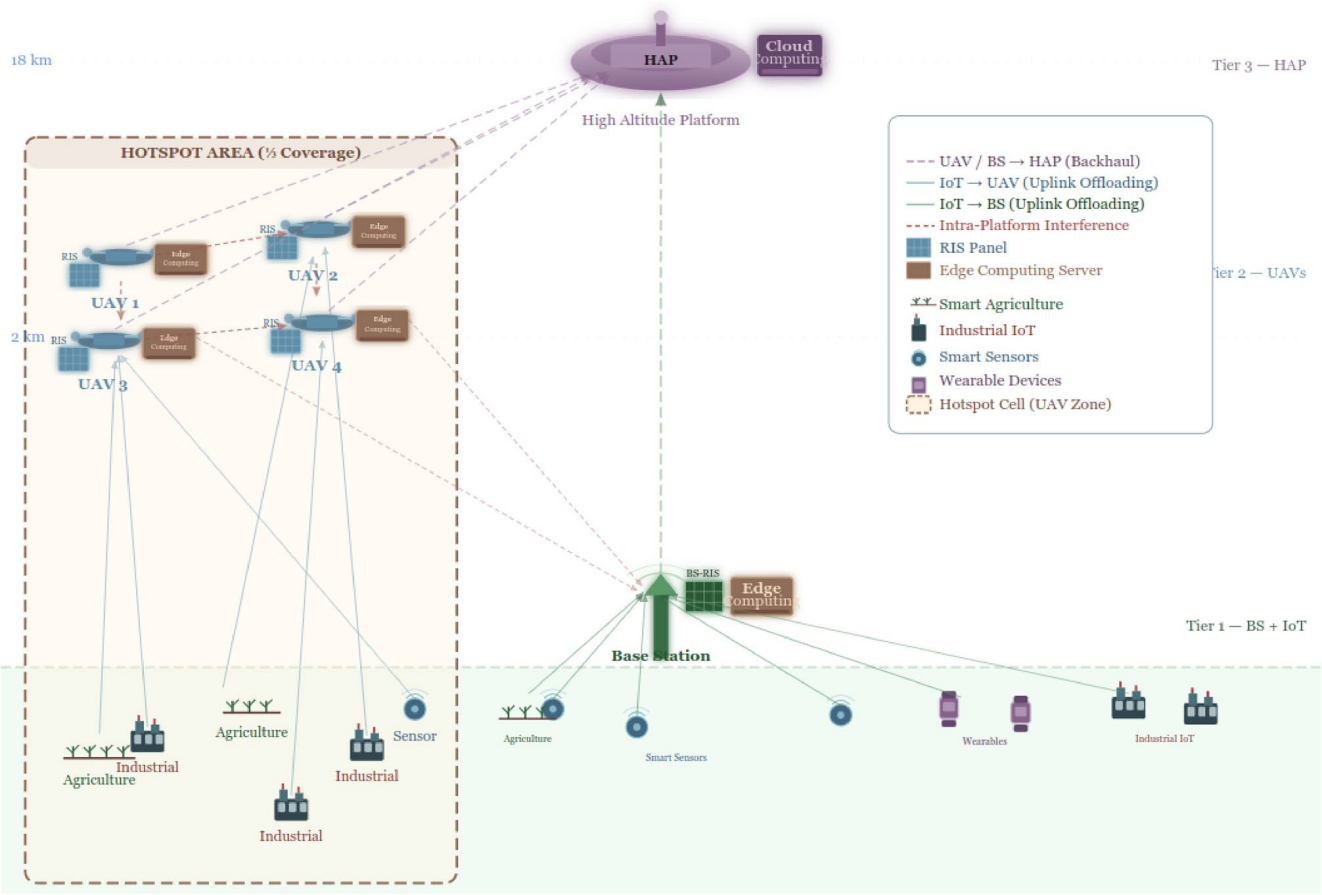
Three-tier RIS-enhanced hierarchical aerial computing network.

### Network architecture

We consider a three-tier heterogeneous aerial computing architecture serving a set $\mathscr {I} = \{1, 2, \ldots , I$} of ground-based Internet of Things (IoT) devices distributed across a target area of $10\,\text {km} \times 10\,\text {km}$. The computing infrastructure comprises three tiers of platforms with fundamentally different operational characteristics.

The first tier is a single ground-based base station *b* equipped with a reconfigurable intelligent surface (RIS) panel of $N_{b}$ elements and a high-capacity edge computing server powered by the electrical grid. The base station is fixed at position $\textbf{q}_{b}$ at ground level and provides persistent coverage for ground-proximate IoT devices. Here, *persistent coverage* refers to system-level service continuity rather than uninterrupted UAV operation: the grid-powered BS-RIS tier provides continuous, energy-unconstrained service for ground-proximate IoT devices independently of UAV battery state, while a staggered UAV rotation schedule ensures that at least 3 of the 4 UAVs remain operational at any time during battery replenishment cycles, maintaining approximately 75% of peak hotspot capacity during transitions. The cell radius of approximately $4\,\text {km}$^3^, offering unlimited energy and computational resources two to five times more powerful than UAV payloads. The second tier consists of a set $\mathscr {U} = \{1, 2, \ldots , U\}$, each equipped with an onboard RIS panel of $N_{u}$ passive reflecting elements and a mobile edge computing server. UAV *u* is deployed at a fixed horizontal position $\textbf{q}_{u}$ and altitude $H_{u} = 2\,\text {km}$, concentrating coverage in the hotspot sub-region (left one-third of the deployment area) where high-density IoT traffic originates from smart agriculture and industrial IoT applications. UAVs operate on rechargeable batteries with a finite energy budget $E_{u} = 100\,\text {kJ}$, and their onboard RIS is configured to simultaneously enhance desired signal gain and suppress co-channel interference via phase optimization. The third tier is a single high-altitude platform (HAP) *h* positioned at stratospheric altitude $H_{\textrm{HAP}} = 18\,\text {km}$, directly above the centre of the deployment area. The HAP serves as a high-capacity computational reservoir with processing capability $C = 3 \times 10^{10}\,\text {cycles/s}$, receiving overloaded tasks from both UAVs and the base station via dedicated backhaul links. Owing to its wide footprint, the HAP covers the entire deployment area but does not serve as a direct access point for IoT devices; it accepts tasks only when properly relayed through the edge tier. The three-tier network architecture is illustrated in Fig. 1. This paper extends the UAV–HAP framework from our prior work^2^ by integrating the base station tier and introducing explicit co-channel interference management among IoT-to-platform uplinks.

### Channel model

Each UAV and BS is equipped with a reconfigurable intelligent surface comprising *N* passive reflecting elements which selected over active relay-based alternatives for three system-level reasons specific to the proposed deployment^12,16,17^: (i) active relays consume 8–12$\times$ more power than passive RIS for comparable coverage gains^12^, unacceptably reducing UAV flight endurance and computational capacity for battery-constrained aerial platforms; (ii) the sub-array architecture exploits $N=256$ passive elements to simultaneously perform signal enhancement and interference suppression, dual functions that would require separate active hardware in relay-based designs; and (iii) the grid-powered BS already provides the persistent high-power transmission capability that active relays are typically deployed to supply, eliminating the primary motivation for relay deployment in the considered three-tier architecture.

The choice of $N=256$ elements per panel balances array gain and computational complexity. Each sub-array of $N_s=64$ elements provides an array gain of $N_s^2=4096$, sufficient for the considered $10\,\text {km}\times 10\,\text {km}$ deployment area at $f_c=2.4$ GHz, while the total RCG complexity $\mathscr {O}((|\mathscr {U}|+1) K_{\textrm{RCG}}N^2)$ remains tractable within the 1.46 s execution budget. Increasing *N* beyond 256 yields diminishing SINR returns while introducing quadratic growth in optimization time, as confirmed by the parametric analysis presented in Section "Results and discussion" , N partitioned into $M = 4$ non-overlapping sub-arrays folow directly from the network topology: each UAV faces exactly 4 interfering neighbours (3 other UAVs + 1 BS), and the BS faces all 4 UAVs, so dedicating one sub-array per interferer achieves complete interference coverage without redundancy. With $N=256$ elements, $M=4$ yields $N/M=64$ elements per sub-array, providing sufficient spatial degrees of freedom for the target 85% suppression ($\psi ^\textrm{sup}=0.15$), as validated in Section "Results and discussion". The HAP does not carry an RIS panel; it serves purely as a high-capacity cloud computing reservoir receiving offloaded tasks over dedicated backhaul links.

#### IoT to UAV-RIS link

The channel gain between IoT device *i* and UAV-RIS *u* is^18^:

1 $$\begin{aligned} G_{i,u}^{\text {RIS}} = G_{i,u} \cdot G^{\text {RIS}}_u, \end{aligned}$$

where

2 $$\begin{aligned} G_{i,u} = \frac{G_0}{\Vert \textbf{q}_u - \textbf{q}_i\Vert ^2 + H_u^2} \end{aligned}$$

is the line-of-sight channel gain between IoT *i* and UAV *u* (I2U), $G_0$ is the reference channel gain at unit distance, $\textbf{q}_u$ and $\textbf{q}_i$ are the horizontal positions of UAV *u* and IoT *i*, respectively, $H_u = 2$ km is the UAV altitude, and the aggregate RIS gain of UAV *u* accounts for the sub-array partitioning^8^:

3 $$\begin{aligned} G^{\text {RIS}}_u = G_{\text {orient}}(\theta _u) \cdot \left| \sum _{n=1}^{N} \alpha _n^u(\phi _n^u) e^{j\phi _n^u} \right| ^2, \end{aligned}$$

where $G_{\textrm{orient}}(\theta _u)$ is the orientation gain factor, $\alpha ^u_n(\varphi ^u_n) \in [0,1]$ is the phase-dependent amplitude reflection coefficient of the *n*-th element, and $\varphi ^u_n \in [0,2\pi )$ is its phase shift. The *N* elements are partitioned into $M = 4$ non-overlapping sub-arrays $\{\mathscr {S}^u_m\}_{m=1}^{4}$, each of size *N*/*M*, where sub-array *m* is assigned to suppress interference from platform *m* (the remaining three UAVs and the BS).

#### Interference-aware SINR at UAV

Because all UAVs and the BS share the same spectrum and provide overlapping coverage of the entire area, each UAV receiver is exposed to both intra-UAV co-channel interference (from devices sharing the same sub-channel on UAV *u*) and inter-platform interference (from transmissions associated with other UAVs and the BS). The SINR for device *i* served by UAV *u* on sub-channel *k* is^7,19^:

4 $$\begin{aligned} \textrm{SINR}_{i,u} = \frac{P^{\textrm{tr}}_i\, G^{\textrm{RIS}}_{i,u}}{\sigma ^2 + \underbrace{I^{\textrm{intra}}_{i,u}}_{\text {intra-UAV CCI}} + \underbrace{I^{\textrm{inter}}_{i,u}}_{\text {inter-platform}}}, \end{aligned}$$

where $\sigma ^2$ is the noise power density and $P_{i}^\textrm{tr}$ is the transmission power from IoT *i* to UAV *u*.

The *intra-UAV* co-channel interference is caused by all devices matched to UAV *u* on the same sub-channel *k*:

5 $$\begin{aligned} I^{\textrm{intra}}_{i,u} = \sum _{\begin{array}{c} j \in \mathscr {S}^u_k,\; j \ne i \end{array}} P^{\textrm{tr}}_j\, G^{\textrm{RIS}}_{j,u} \cdot A_u^j, \end{aligned}$$

where $\mathscr {S}^u_k$ is the set of devices assigned to sub-channel *k* on UAV *u*, and $A_u^j \in \{0,1\}$ indicates whether IoT device *j* is associated with UAV *u*. The interference term $\sum _{j \ne i} P_j^\textrm{tr} G_{j,u}^\textrm{RIS} \cdot A_u^j$ captures *intra-UAV co-channel interference*: only IoT devices matched to the same UAV *u* (where $A_u^j = 1$) and assigned to the same sub-channel contribute to interference.

The *inter-platform* interference aggregates signals from all other UAVs $u' \ne u$ and from the BS, attenuated by the suppression achieved by the dedicated sub-arrays:

6 $$\begin{aligned} I^{\textrm{inter}}_{i,u} = \sum _{u' \ne u} \psi ^{\textrm{sup}}_{u,u'}\, P^{\textrm{tr}}_{u'} G^{\textrm{RIS}}_{i,u'} + \psi ^{\textrm{sup}}_{u,b}\, P^{\textrm{tr}}_b\, G^{\textrm{RIS}}_{i,b}, \end{aligned}$$

where $\psi ^{\textrm{sup}}_{u,u'} \in [0,1]$ and $\psi ^{\textrm{sup}}_{u,b} \in [0,1]$ are the residual interference fractions after sub-array suppression targeting UAV $u'$ and the BS, respectively. A value of $\psi = 0$ corresponds to perfect cancellation, while $\psi = 1$ indicates no suppression.

The interference-aware data rate is formulated as^6,20,21^:

7 $$\begin{aligned} R_{i,u} = \textrm{BW}_{iu}\,\log _2\!\left( 1 + \textrm{SINR}_{i,u}\right) , \end{aligned}$$

where $BW_{iu}$ is the bandwidth of the IoT-to-UAV channel.

#### IoT to BS-RIS link

The ground-to-ground channel between IoT device *i* and the BS follows a distance-dependent path loss model with a higher path loss exponent $\gamma \in [2.5,\,4]$ reflecting terrestrial multipath and obstruction effects^22^:

8 $$\begin{aligned} G^{\textrm{RIS}}_{i,b} = G_{i,b} \cdot G^{\textrm{RIS}}_{b}, \end{aligned}$$

where $G_{i,b} = G_0 / d^{\gamma }_{i,b}$ represents the distance-dependent path loss with $d_{i,b} = \Vert \textbf{q}_b - \textbf{q}_i\Vert$ being the horizontal distance between IoT *i* and BS, $\textbf{q}_b$ is the BS horizontal position, and $\gamma$ is the path loss exponent .

The BS-RIS gain is:

9 $$\begin{aligned} G^{\textrm{RIS}}_{b} = G_{\textrm{orient}}(\theta _b) \Bigg |\sum _{n=1}^{N_b} \alpha ^b_n(\varphi ^b_n)\,e^{j\varphi ^b_n}\Bigg |^2, \end{aligned}$$

where $N_b$ is the number of RIS elements on the BS, $\alpha _n^b$ and $\phi _n^b$ are the amplitude and phase shift of the *n*-th element of the BS-RIS, respectively. The BS-RIS elements are similarly partitioned into $M = 4$ sub-arrays, one per UAV, each configured to suppress the interference signal arriving from the corresponding UAV.

#### Interference-aware SINR at the BS

The SINR for device *i* associated with the BS on sub-channel *k* is:

10 $$\begin{aligned} \textrm{SINR}_{i,b} = \frac{P^{\textrm{tr}}_i\, G^{\textrm{RIS}}_{i,b}}{\sigma ^2 + \underbrace{I^{\textrm{intra}}_{i,b}}_{\text {intra-BS CCI}} + \underbrace{I^{\textrm{inter}}_{i,b}}_{\text {inter-platform}}} \end{aligned}$$

The *intra-BS* co-channel interference from devices sharing sub-channel *k* at the BS is:

11 $$\begin{aligned} I^{\textrm{intra}}_{i,b} = \sum _{\begin{array}{c} j \in \mathscr {S}^b_k,\; j \ne i \end{array}} P^{\textrm{tr}}_j\, G^{\textrm{RIS}}_{j,b} \end{aligned}$$

The *inter-platform* interference at the BS originates from all four UAVs, suppressed by the corresponding BS sub-arrays:

12 $$\begin{aligned} I^{\textrm{inter}}_{i,b} = \sum _{u \in \mathscr {U}} \psi ^{\textrm{sup}}_{b,u}\, P^{\textrm{tr}}_u\, G^{\textrm{RIS}}_{i,u}, \end{aligned}$$

where $\psi ^{\textrm{sup}}_{b,u} \in [0,1]$ is the residual fraction of UAV *u*’s interference after BS sub-array suppression. The achievable data rate for the IoT-to-BS link is:

13 $$\begin{aligned} R_{i,b} = \textrm{BW}_{ib}\,\log _2\!\left( 1 + \textrm{SINR}_{i,b}\right) = \textrm{BW}_{ib}\,\log _2\!\left( 1 + \frac{P^{\textrm{tr}}_i\, G^{\textrm{RIS}}_{i,b}}{\sigma ^2 + I^{\textrm{intra}}_{i,b} + I^{\textrm{inter}}_{i,b}}\right) , \end{aligned}$$

where $BW_{ib}$ is the allocated bandwidth for the IoT-BS link. A critical observation is that the BS-RIS phase vector $\boldsymbol{\varphi }^b$ simultaneously influences the desired gain $G^{\textrm{RIS}}_{i,b}(\boldsymbol{\varphi }^b)$, the intra-BS interference terms $\{G^{\textrm{RIS}}_{j,b}(\boldsymbol{\varphi }^b)\}_{j \in \mathscr {S}^b_k}$, and the suppression factors $\{\psi ^{\textrm{sup}}_{b,u}\}_{u \in \mathscr {U}}$.

#### Backhaul links (UAV/BS to HAP)

The backhaul links from UAV-RIS *u* and the BS-RIS to the HAP follow free-space propagation^23,24^. The achievable data rate for platform $p \in \{u, b\}$ is:

14 $$\begin{aligned} R_p = BW_p \cdot \log _2\left( 1 + \frac{P_p^{\text {tr}} \, G_p \, L_s \, L_l \cdot G_p^{\text {RIS}}}{k_B T_s BW_p}\right) , \quad p \in \{u, b\} \end{aligned}$$

where $BW_p$ is the bandwidth of the backhaul channel, $P_p^{\text {tr}}$ is the transmission power of platform *p*, $G_p$ is the antenna power gain, $L_s = \left( \frac{c}{4\pi d_p f_p}\right) ^2$ is the free-space path loss, $d_p$ is the platform-to-HAP distance, $f_p$ is the backhaul center frequency, $L_l$ is the atmospheric attenuation loss, $k_B$ is the Boltzmann constant, and $T_s$ denotes the system noise temperature. For the BS ($p = b$), the distance is $d_b = \sqrt{\Vert \textbf{q}_b - \textbf{q}_h\Vert ^2 + H^2}$, where $\textbf{q}_h$ is the HAP horizontal position and $H = 18$ km is the HAP altitude. For UAV *u* ($p = u$), $d_u$ is the UAV-to-HAP distance. The BS benefits from higher transmission power ($P_b^{\text {tr}} = 15$ W) enabled by grid power availability, potentially offering lower and more stable backhaul delays compared to battery-constrained UAV platforms.

### Time cost model

The total time cost for completing an IoT task consists of transmission delays and computation processing times across the hierarchical aerial computing framework, now extended to include the BS-RIS platform.

#### Time cost for transmission

**IoT to UAV:** The time required to transmit data from IoT device *i* to UAV *u* is given by^25^:

15 $$\begin{aligned} T_{iu} = \frac{K_i A_i^u}{R_{i,u}}, \end{aligned}$$

where $K_i$ represents the data size of IoT *i*, $A_i^u \in \{0,1\}$ indicates whether the task of IoT $i \in \mathscr {I}$ is offloaded to UAV *u*, and $R_{i,u}$ denotes the achievable data rate between IoT *i* and UAV *u* from Eq. (7).

*IoT to BS* The time required to transmit data from IoT device *i* to BS-RIS is:

16 $$\begin{aligned} T_{ib} = \frac{K_i A_i^b}{R_{i,b}}, \end{aligned}$$

where $A_i^b \in \{0,1\}$ indicates whether the task of IoT *i* is offloaded to BS-RIS, and $R_{i,b}$ is the achievable data rate from Eq. (13). The ground-to-ground propagation model for BS links may result in different transmission times compared to aerial UAV links, particularly for IoT devices in urban or obstructed environments where the BS provides more reliable connectivity.

*UAV to HAP* When a UAV relays IoT data to HAP for computation, the transmission time is calculated using^2^:

17 $$\begin{aligned} T_u = \frac{K_i B_{i,u}}{R_u}, \end{aligned}$$

where $B_{i,u} \in \{0,1\}$ indicates whether the task from IoT *i* is forwarded to HAP by UAV *u*, and $R_b$ is given by Eq.(14).

*BS to HAP* When the BS-RIS relays IoT data to HAP, the backhaul transmission time is:

18 $$\begin{aligned} T_b = \frac{K_i B_{i,b}}{R_b}, \end{aligned}$$

where $B_{i,b} \in \{0,1\}$ indicates whether the task from IoT *i* is forwarded to HAP by BS, and $R_b$ is the BS-to-HAP data rate from Eq. (14). The BS benefits from higher transmission power and fixed positioning, potentially offering lower and more stable backhaul delays compared to mobile UAV platforms.

#### Time cost for computing

*UAV computing time* The time for UAV *u* to complete the computation task for IoT *i* is expressed as^2^:

19 $$\begin{aligned} T_i^u = \frac{K_i \alpha _i^u}{\frac{C_u}{\rho _u}} = \frac{K_i \alpha _i^u \rho _u}{C_u}, \end{aligned}$$

where $\alpha _i^u \in \{0,1\}$ indicates whether the task of IoT $i \in \mathscr {I}$ is computed by UAV *u*, $\rho _u$ represents the computational resource cost per bit (in cycles/bit), and $C_u$ denotes the computation capability of UAV *u* (in cycles/s).

*BS computing time* The time for BS to complete the computation task for IoT *i* is:

20 $$\begin{aligned} T_i^b = \frac{K_i \alpha _i^b \rho _b}{C_b}, \end{aligned}$$

where $\alpha _i^b \in \{0,1\}$ indicates whether the task of IoT *i* is computed by BS, $\rho _b$ represents the computational resource cost per bit for BS (same as UAVs, $\rho _b = \rho _u = 270$ cycles/bit for homogeneous task complexity), and $C_b$ denotes the computation capability of BS ($C_b = 2 \times 10^9$ cycles/s). The BS’s grid-powered infrastructure enables higher computational capacity compared to battery-constrained UAVs ($C_u = 10^9$ cycles/s), resulting in lower processing times for tasks computed locally at the BS.

*HAP computing time* Similarly, the computation time at the HAP is given by^2^:

21 $$\begin{aligned} T_i = \frac{K_i \delta _i}{C/\mu } = \frac{K_i \delta _i \mu }{C}, \end{aligned}$$

where $\delta _i \in \{0,1\}$ indicates whether the task of IoT $i \in \mathscr {I}$ is computed by HAP, $\mu$ is the computational resource cost per bit for the HAP (in cycles/bit), and *C* represents the HAP’s computation capability (in cycles/s).

*Total time cost* The total time cost for IoT device *i* encompasses all potential paths through the hierarchical system:

22 $$\begin{aligned} \begin{aligned} T_i^{\text {total}}&= \sum _{u \in \mathscr {U}} \left[ \frac{K_i A_i^u}{R_{i,u}} + \frac{K_i \alpha _i^u \rho _u}{C_u} + \frac{K_i B_{i,u}}{R_u} \right] + \frac{K_i A_i^b}{R_{i,b}} + \frac{K_i \alpha _i^b \rho _b}{C_b} + \frac{K_i B_{i,b}}{R_b} + \frac{K_i \delta _i \mu }{C} \end{aligned} \end{aligned}$$

This comprehensive model accounts for all transmission and computation delays in the three-tier hierarchical system. The first term represents the UAV path (IoT-to-UAV transmission, UAV computation, and UAV-to-HAP relay), while the second terms account for the BS-RIS path (IoT-to-BS transmission, BS computation, and BS-to-HAP relay). The final term represents HAP computation, which may serve tasks offloaded from either UAVs or BS. The end-to-end delay constraint $T_i^{\text {total}} \le D_i$ must be satisfied for successful task completion, where $D_i$ is the maximum tolerable delay for IoT device *i*.

### Energy cost model

The energy consumption in the hierarchical aerial computing framework consists of operational energy and task-specific energy costs for IoT devices, UAVs, BS-RIS, and HAPs. Each entity has distinct energy consumption patterns based on its computational and communication activities.

#### Energy cost of IoT

The total energy cost $E_i^c$ of IoT device *i* comprises the basic operational energy and transmission energy for data offloading^26,27^:

23 $$\begin{aligned} E_i^c = E_i^o + \sum _{u \in \mathscr {U}} \frac{P_i^{\text {tr}} K_i A_i^u}{R_{i,u}} + \frac{P_i^{\text {tr}} K_i A_i^b}{R_{i,b}}, \end{aligned}$$

where $E_i^o$ represents the basic operational energy cost of IoT *i*, $P_i^{\text {tr}}$ denotes the transmission power from IoT *i*, $K_i$ is the data size, $R_{i,u}$ and $R_{i,b}$ represent the achievable data rates to UAV *u* and BS, respectively. The second term accounts for transmission energy to any matched UAV, while the third term accounts for transmission energy to the BS-RIS platform. Note that each IoT device is matched to at most one edge platform (UAV or BS) due to the constraint $\sum _{u \in \mathscr {U}} A_i^u + A_i^b \le 1$, ensuring only one of these transmission energy terms is active for each device.

#### Energy cost of UAV

The total energy cost $E_u^c$ of UAV *u* includes basic operation, computation processing, data transmission to HAP, and RIS operation^2^:

24 $$\begin{aligned} E_u^c = E_u^o + \sum _{i \in \mathscr {I}} \zeta _u C_u^2 K_i \rho _u \alpha _i^u + \sum _{i \in \mathscr {I}} \frac{P_u^{\text {tr}} K_i B_{i,u}}{R_u} + E_u^{\text {RIS}}, \end{aligned}$$

where $E_u^o$ is the basic operational energy cost (e.g., hovering energy), $\zeta _u$ represents the energy consumption coefficient depending on the UAV’s processor chip architecture, $C_u$ is the computing capability of UAV, $\rho _u$ is the computational resource cost per bit, $P_u^{\text {tr}}$ is the transmission power from UAV to HAP, $B_{i,u}$ represents the data forwarding decision, $R_u$ is the UAV-to-HAP data rate, and $E_u^{\text {RIS}}$ accounts for the energy costs of operating the reconfigurable intelligent surface.

#### Energy cost of BS

The total energy cost $E_b^c$ of the BS-RIS includes basic operation, computation processing, data transmission to HAP, and RIS operation:

25 $$\begin{aligned} E_b^c = E_b^o + \sum _{i \in \mathscr {I}} \zeta _b C_b^2 K_i \rho _b \alpha _i^b + \sum _{i \in \mathscr {I}} \frac{P_b^{\text {tr}} K_i B_{i,b}}{R_b} + E_b^{\text {RIS}}, \end{aligned}$$

where $E_b^o$ is the basic operational energy cost of the BS (including cooling, control systems, and RIS circuitry), $\zeta _b$ represents the energy consumption coefficient for the BS processor (typically $\zeta _b = \zeta _u = 10^{-28}$ for similar processor architectures), $C_b$ is the computing capability of BS ($C_b = 2 \times 10^9$ cycles/s), $\rho _b$ is the computational resource cost per bit (same as UAVs, $\rho _b = \rho _u = 270$ cycles/bit), $P_b^{\text {tr}}$ is the transmission power from BS to HAP ($P_b^{\text {tr}} = 15$ W, higher than UAVs due to grid power availability), $B_{i,b}$ indicates whether task *i* is forwarded to HAP by BS, $R_b$ is the BS-to-HAP backhaul data rate, and $E_b^{\text {RIS}}$ accounts for RIS operation energy.

Unlike battery-constrained UAVs, the BS is connected to the electrical grid, providing effectively unlimited energy supply ($E_b^c \le E_b$ where $E_b \rightarrow \infty$ in practice). However, we model BS energy consumption for completeness and to evaluate system-wide energy efficiency. In practical deployments, BS operational costs are dominated by grid electricity pricing rather than energy availability constraints. This grid-powered advantage enables the BS to sustain higher computational loads and transmission power levels without the energy budget limitations faced by aerial platforms.

#### Energy cost of HAP

The total energy cost $E^c$ of the HAP system primarily consists of basic operational energy and computation energy^2^:

26 $$\begin{aligned} E^c = E^o + \sum _{i \in \mathscr {I}} \zeta C^2 K_i \delta _i \mu , \end{aligned}$$

where $E^o$ represents the basic operational energy cost of the HAP, $\zeta$ is the energy consumption coefficient for the HAP’s processor, *C* is the computing capability of HAP, $\delta _i$ indicates whether task *i* is computed by HAP (regardless of whether it was relayed from UAV or BS), and $\mu$ represents the computation resource cost per bit for HAP. These energy models ensure that the hierarchical aerial computing system operates within the energy budget constraints of each platform while maximizing the overall system performance.

#### RIS energy cost

The energy consumption of the reconfigurable intelligent surface mounted on each UAV and the BS, denoted as $E_u^{\text {RIS}}$ and $E_b^{\text {RIS}}$ respectively, is modeled as the total energy required to operate the RIS over the platform’s active mission duration. This accounts for both the power dissipated by the individual reflecting elements and the control circuitry responsible for configuring their phase shifts.

For a passive RIS comprising *N* (for UAVs) or $N_b$ (for BS) tunable reflecting elements, the total steady-state power consumption is given by^28^:

27 $$\begin{aligned} & P_u^{\text {RIS}} = N \cdot P_{\text {element}} + P_{\text {ctrl}}^u, \end{aligned}$$

28 $$\begin{aligned} & P_b^{\text {RIS}} = N_b \cdot P_{\text {element}} + P_{\text {ctrl}}^b, \end{aligned}$$

where $P_{\text {element}}$ is the bias power required per reflecting element (typically on the order of milliwatts), and $P_{\text {ctrl}}^u$ and $P_{\text {ctrl}}^b$ are the power consumption of the RIS control units for UAV and BS respectively, which compute and set the phase configurations ${\phi }^u$ and ${\phi }^b$.

The total energy consumed by the RIS over an operational time window $T_{\text {op}}$ is therefore:

29 $$\begin{aligned} & E_u^{\text {RIS}} = P_u^{\text {RIS}} \cdot T_{\text {op}}, \end{aligned}$$

30 $$\begin{aligned} & E_b^{\text {RIS}} = P_b^{\text {RIS}} \cdot T_{\text {op}}, \end{aligned}$$

where $T_{\text {op}}$ represents the active computing/communication mission phase. For UAVs, $T_{\text {op}}$ corresponds to the flight mission duration, while for the BS, it represents the continuous operational period (typically much longer due to grid power availability).

### Problem formulation

The objective is to maximize the total volume of IoT data successfully processed by the hierarchical aerial computing platforms (RIS-equipped UAVs, BS-RIS, and HAP) while managing both intra-platform co-channel interference and inter-platform interference through RIS sub-array suppression and enhancing communication performance through RIS phase optimization. The RIS phase optimization subproblem is formulated as^29^:

31a $$\begin{aligned} \quad \quad \quad \quad \quad \quad \text {(P-RIS):} \quad&\max _{\phi } R_{\text {i,p}}(\phi ) \end{aligned}$$

31b $$\begin{aligned} \text {where} \quad&R_{\text {i.p}}\mathbf {(\phi )} = \sum _{u \in \mathscr {U}} \sum _{i \in \mathscr {I}} BW_{i,u} \cdot \log _2(1 + \text {SINR}_{i,u}), \end{aligned}$$

where $p \in \{\text {UAVs}, \text {BS}\}$ denotes the platform (UAV or BS) and ${\phi }$ represents the phase configuration vector.

The optimization problem is formulated as follows:

32 $$\begin{aligned} \begin{aligned} \text {(P0):} \quad \max _{\begin{array}{c} A^u, A^b, \alpha ^u, \alpha ^b, \\ B^u, B^b, \delta , {\phi }^u, {\phi }^b, {\psi } \end{array}}&\sum _{i \in \mathscr {I}} K_i \left( \sum _{u \in \mathscr {U}} \alpha _i^u + \alpha _i^b + \delta _i \right) \end{aligned} \end{aligned}$$

The formulated problem (P0) is a mixed-integer nonlinear programming (MINLP) problem that exhibits strong coupling between discrete resource allocation decisions and continuous RIS phase configuration variables. The joint MINLP problem is formulated over the decision variables ${A}^u$, ${A}^b$, ${\alpha }^u$, ${\alpha }^b$, ${B}^u$, ${B}^b$, ${\delta }$, ${\varphi }^u$, ${\varphi }^b$, $\boldsymbol{\psi }$ defined in Table 1, where $\textbf{A}^u$ and $\textbf{A}^b$ are IoT-to-UAV and IoT-to-BS association indicators, respectively $\boldsymbol{\alpha }^u$ and $\boldsymbol{\alpha }^b$ are local computation indicators at UAVs and BS, respectively $\textbf{B}^u$ and $\textbf{B}^b$ are UAV-to-HAP and BS-to-HAP offloading indicators respectively, $\boldsymbol{\delta }$ is the HAP computation indicator, $\boldsymbol{\varphi }^u$ and $\boldsymbol{\varphi }^b$ are the RIS phase shift vectors for UAVs and BS, and $\boldsymbol{\psi } = \{\psi ^{\textrm{sup}}_{u,u'}, \psi ^{\textrm{sup}}_{u,b}, \psi ^{\textrm{sup}}_{b,u}\}$ are residual inter-platform interference fractions after sub-array suppression.

#### Subject to:

33a $$\begin{aligned}&\sum _{u \in \mathscr {U}} A_i^u + A_i^b \le 1, \quad \forall i \in \mathscr {I} \end{aligned}$$

33b $$\begin{aligned}&\alpha _i^u + B_{i,u} = A_i^u, \quad \forall i \in \mathscr {I}, u \in \mathscr {U} \end{aligned}$$

33c $$\begin{aligned}&\alpha _i^b + B_{i,b} = A_i^b, \quad \forall i \in \mathscr {I} \end{aligned}$$

33d $$\begin{aligned}&\sum _{i \in \mathscr {I}} A_i^u \le N_u, \quad \forall u \in \mathscr {U} \end{aligned}$$

33e $$\begin{aligned}&\sum _{i \in \mathscr {I}} A_i^b \le N_b \end{aligned}$$

33f $$\begin{aligned}&\delta _i \le \sum _{u \in \mathscr {U}} B_{i,u} + B_{i,b}, \quad \forall i \in \mathscr {I} \end{aligned}$$

34a $$\begin{aligned}&\sum _{i \in \mathscr {I}} \alpha _i^u \rho _u \le C_u, \quad \forall u \in \mathscr {U} \end{aligned}$$

34b $$\begin{aligned}&\sum _{i \in \mathscr {I}} \alpha _i^b \rho _b \le C_b \end{aligned}$$

34c $$\begin{aligned}&\sum _{i \in \mathscr {I}} \delta _i \mu \le C \end{aligned}$$

35a $$\begin{aligned}&E_i^c \le E_i, \quad \forall i \in \mathscr {I} \end{aligned}$$

35b $$\begin{aligned}&E_u^c \le E_u, \quad \forall u \in \mathscr {U} \end{aligned}$$

35c $$\begin{aligned}&E_b^c \le E_b \end{aligned}$$

35d $$\begin{aligned}&E^c \le E \end{aligned}$$

36a $$\begin{aligned}&T_i^{\text {total}} \le D_i, \quad \forall i \in \mathscr {I} \end{aligned}$$

36b $$\begin{aligned}&\sum _{i \in \mathscr {I}} K_i \le \min \left( \sum _{u \in \mathscr {U}} \frac{C_u}{\rho _u} + \frac{C_b}{\rho _b} + \frac{C}{\mu }, \sum _{i \in \mathscr {I}} K_i \cdot \mathbb {1}(E_i^{\text {tr}} \le E_i) \right) \end{aligned}$$

37a $$\begin{aligned}&\textrm{SINR}_{i,u} \ge \Gamma _{\min }, \quad \forall i\in \mathscr {I}_u,\; u\in \mathscr {U} \end{aligned}$$

37b $$\begin{aligned}&\textrm{SINR}_{i,b} \ge \Gamma _{\min }, \quad \forall i \in \mathscr {I}_b \end{aligned}$$

38a $$\begin{aligned}&\sum _{n=1}^{N} |\alpha _n^u|^2 \le N, \quad \forall u \in \mathscr {U} \end{aligned}$$

38b $$\begin{aligned}&\sum _{n=1}^{N_b} |\alpha _n^b|^2 \le N_b \end{aligned}$$

38c $$\begin{aligned}&\phi _n^u \in [0, 2\pi ], \quad \forall n \in \{1,2,...,N\}, u \in \mathscr {U} \end{aligned}$$

38d $$\begin{aligned}&\phi _n^b \in [0, 2\pi ], \quad \forall n \in \{1,2,...,N_b\} \end{aligned}$$

38e $$\begin{aligned}&0 \le \alpha _n^u \le 1, \quad \forall n \in \{1,2,...,N\}, u \in \mathscr {U} \end{aligned}$$

38f $$\begin{aligned}&0 \le \alpha _n^b \le 1, \quad \forall n \in \{1,2,...,N_b\} \end{aligned}$$

38g $$\begin{aligned}&|e^{j\phi _n^u}| = 1, \quad \forall n \in \{1,2,...,N\}, u \in \mathscr {U} \end{aligned}$$

38h $$\begin{aligned}&|e^{j\phi _n^b}| = 1, \quad \forall n \in \{1,2,...,N_b\} \end{aligned}$$

39a $$\begin{aligned}&A_i^u, \alpha _i^u, B_{i,u} \in \{0,1\}, \quad \forall i \in \mathscr {I}, u \in \mathscr {U} \end{aligned}$$

39b $$\begin{aligned}&A_i^b, \alpha _i^b, B_{i,b} \in \{0,1\}, \quad \forall i \in \mathscr {I} \end{aligned}$$

39c $$\begin{aligned}&\delta _i \in \{0,1\}, \quad \forall i \in \mathscr {I} \end{aligned}$$

40a $$\begin{aligned}&\psi ^{\textrm{sup}}_{u,u'} \in [0,1], \quad \forall u,u'\in \mathscr {U},\; u\ne u' \end{aligned}$$

40b $$\begin{aligned}&\psi ^{\textrm{sup}}_{u,b} \in [0,1], \quad \forall u\in \mathscr {U} \end{aligned}$$

40c $$\begin{aligned}&\psi ^{\textrm{sup}}_{b,u} \in [0,1], \quad \forall u\in \mathscr {U} \end{aligned}$$

41a $$\begin{aligned}&\bigcup _{m=1}^{4}\mathscr {S}^u_m = \{1,\ldots ,N\},\quad \mathscr {S}^u_m \cap \mathscr {S}^u_{m'} = \emptyset ,\; m \ne m',\quad \forall u\in \mathscr {U} \end{aligned}$$

41b $$\begin{aligned}&|\mathscr {S}^u_m| = N/4, \quad \forall m\in \{1,2,3,4\},\; u\in \mathscr {U} \end{aligned}$$

41c $$\begin{aligned}&\bigcup _{m=1}^{4}\mathscr {S}^b_m = \{1,\ldots ,N_b\},\quad \mathscr {S}^b_m \cap \mathscr {S}^b_{m'} = \emptyset ,\; m \ne m' \end{aligned}$$

41d $$\begin{aligned}&|\mathscr {S}^b_m| = N_b/4, \quad \forall m\in \{1,2,3,4\} \end{aligned}$$

The constraints of problem (P0) can be categorized into nine groups based on their functional roles in the hierarchical aerial computing system:

*Association and flow control constraints* Constraints (33a–33f) establish the fundamental communication and data flow rules. The exclusive association constraint (33a) ensures each IoT device connects to at most one edge platform (UAV or BS), preventing communication conflicts and enabling effective RIS beamforming. The flow conservation constraints (33b) and (33c) enforce that tasks offloaded to an edge platform must be either computed locally or forwarded to HAP, maintaining logical consistency in the hierarchical structure. The capacity constraints (33d) and (33e) model the physical limitations on simultaneous connections each platform can handle. The HAP forwarding constraint (33f) ensures HAP only processes tasks properly relayed through the edge tier (UAV or BS), preserving the hierarchical architecture where HAP serves as a cloud-tier backup rather than a direct access point.

*Computational resource constraints* Constraints (34a–34c) enforce finite computational capacities across all network entities. Constraint (34a) ensures the total computational workload assigned to each UAV *u* does not exceed its processing capability $C_u$, where $\alpha _i^u$ indicates local UAV computation and $\rho _u$ represents the computational cost per bit. Similarly, constraint (34b) enforces the BS computational capacity limit $C_b = 2 \times 10^9$ cycles/s, which is double the UAV capacity due to grid-powered infrastructure advantages. Constraint (34c) ensures the aggregate load offloaded to HAP remains within its high-capacity processing capability *C*. These constraints are fundamentalo syst feasibility and prevent overloading of processing units.

*Resource availability constraints* Constraints (35a–35d) enforce finite energy budgets across all network entities. Unlike traditional systems with fixed communication costs, the RIS-enhanced formulation creates dynamic energy consumption patterns where improved channel gains can reduce IoT transmission energy (constraint (35a)) while introducing additional platform energy overhead for RIS operation. Constraint (35b) enforces strict battery limitations for aerial platforms ($E_u = 100$ kJ typical), while constraint (35c) represents the BS energy budget, which is effectively unlimited ($E_b \rightarrow \infty$) due to grid connection but is included for modeling completeness. Constraint (35d) ensures HAP operates within its large but finite energy budget. These constraints ensure sustainable operation within the power limitations of battery-powered devices and aerial platforms.

**Table 1:** Notation list.

Notation	Parameters	Notation	Parameters
$\mathscr {U}$	UAV set, $u \in \mathscr {U}$	$K_i$	Data size of IoT $i \in \mathscr {I}$.
$\mathscr {I}$	IoT user set, $i \in \mathscr {I}$.	$D_i$	IoT delay tolerance.
$\rho _u$ , $\mu$	Computation resource cost of UAV , HAP.	$\delta$	HAP-based computation indicators.
$C_u$ ,*C*	Computing capability of UAV, HAP.	$\sigma ^2$	Noise power density.
$q_u$ , $q_i$	Horizon location of UAV , IoT.	$G_0$	Reference channel gain.
$N_u$	The maximum number of IoT a UAV can serve.	$\tau _0$	Initial step.
$T_{iu}$	Time cost to transmit the data of I2U.	$\rho$	Backtracking factor.
$T_{u}$	Time cost to transmit the data to HAP by UAV *u*.	$c_1$	Armijo parameter
$T_u^i$	Time cost by UAV to complete the computation.	$\varepsilon$	Convergence tolerance
$T^i$	Time cost by HAP to complete the computation.	$\psi ^{\textrm{sup}}$	Residual interference fraction.
$P_i^{tr}$	Transmission power of I2U.	$\alpha _n$	Amplitude reflection coefficients .
$P_u^{tr}$	Transmission power of U2H.	$\lambda _1, \lambda _2, \lambda _3$	Matching weights.
$\zeta _u$ , $\zeta$	Energy consumption coefficient of UAV, HAP.	$w_1, w_2$	Heuristic weights .
$E_i^c$ , $E_u^c$ , $E^c$	Total energy cost of IoT, UAV, HAP.	$L_l$	Atmospheric attenuation.
$E_i^o$ , $E_u^o$ , $E^o$	Basic operation energy cost of IoT, UAV, HAP.	$T_s$	System noise temp.
$E_i$, $E_u$, *E*	Energy budget of IoT, UAV, HAP.	$k_B$	Boltzmann constant.
$E_i^{tr}$	Energy cost for I2U data transmission.	$BW_{iu}$	Bandwidth of I2U channel.
$E_u^{tr}$	Energy cost for U2H data transmission.	$BW_{u}$	Bandwidth of U2H channel.
$E_u^{co}$	Energy cost for computation of *u*.	$R_{i,u}$	Data rate of I2U channel.
$E^{co}$	Energy cost of HAP for computation.	$R_u$	Data rate of U2H channel.
$M_1$	Matching in Algorithm 1.	$A_u^i$, $B^i$, $\alpha _u^i$, $\delta ^i$	Binary variables.
$A^u$	IoT-to-UAV association indicators.	$A^b$	IoT-to-BS association indicators.
$\alpha ^u$	UAV-based computation indicators.	$\alpha ^b$	BS-based computation indicators.
$B^u$	UAV-to-HAP offloading indicators.	$B^b$	BS-to-HAP offloading indicators.
${\phi }^u$	UAV-RIS phase shift vectors.	${\phi }^b$	BS-RIS phase shift vector.
$H_u$	UAV *u* altitude.	$H_{\textrm{HAP}}$	HAP altitude.
*N*	RIS elements per UAV.	$N_b$	RIS elements at BS.
*M*	Sub-arrays per RIS panel.	*N*/*M*	Elements per sub-array.
$\gamma$	Ground path loss exponent.	$G_{\textrm{ant}}$	Antenna gain.
$f_{uh}$	U2H Center Frequency.	$f_{bh}$	BS-to-HAP frequency.
$K_{\textrm{ch}}$	Sub-channels per platform.	$K_{\textrm{RCG}}$	Max RCG iterations.

*Quality of service constraints* Constraint (36a) guarantees that all accepted tasks complete within their specified delay deadlines $D_i$. The RIS optimization directly influences this constraint through enhanced data rates $R_{i,u}({\phi }^u)$ and $R_{i,b}({\phi }^b)$ from Eqs. (7) and (13), potentially allowing the system to serve delay-sensitive applications that would be infeasible in traditional hierarchical systems without intelligent surface enhancement. The total delay $T_i^{\text {total}}$ from Eq. (22) encompasses transmission times (IoT$\rightarrow$UAV/BS, UAV/BS$\rightarrow$HAP) and computation times at all three tiers. The upper bound constraint (36b) establishes a theoretical limit on total computed data based on aggregate computational capacity across all tiers (UAVs, BS, and HAP) and the number of IoT devices with sufficient energy for task offloading, where $\mathbb {1}(\cdot )$ is the indicator function and $E_i^{\text {tr}}$ represents the transmission energy cost.

*SINR feasibility constraints* Constraints (37a–37b) are necessitated by the introduction of inter-platform interference under shared-spectrum operation. All platforms must guarantee a minimum received SINR of $\Gamma _{\min }$ for reliable decoding. The UAV SINR in (37a) includes both intra-UAV CCI and inter-platform interference from other UAVs and the BS, as defined in (4). To enforce reliable communication at the BS tier under shared sub-channel operation, an SINR feasibility constraint is introduced for all IoT devices associated with the BS. This constraint ensures that the received signal quality at the BS remains above a minimum threshold $\Gamma _{\min }$ required for successful decoding in constraint  (37b) where $\mathscr {I}_b = \{i: A_i^b = 1\}$ is the set of IoT devices associated with the BS, and $\textrm{SINR}_{i,b}$ is defined in (10). This constraint directly couples the BS-RIS phase optimization with the device association decisions, as the phase vector $\boldsymbol{\phi }^b$ must be configured to simultaneously satisfy the SINR requirements of all BS-associated devices while managing intra-BS co-channel interference.

*RIS physical constraints* Constraints (38a–38h) are fundamental to modeling the practical electromagnetic behavior of passive RIS on both UAVs and BS. They ensure optimization solutions are physically realizable with standard RIS hardware. The amplitude constraints (38a) and (38b) limit the total reflected power, defining the characteristic of ideal, purely passive RIS where each element does not amplify signals but only shifts phases. The phase range constraints (38c) and (38d) reflect finite tuning resolution of practical phase shifters within $[0, 2\pi ]$. The amplitude reflection coefficient constraints (38e) and (38f) model material imperfections and circuit dissipation, where $\alpha _n^u, \alpha _n^b < 1$ accounts for signal attenuation in real-world metasurfaces. The unit modulus constraints (38g) and (38h) ensure passive reflection (no amplification), conserving energy with $|e^{j\phi _n}| = 1$ exactly.

*Binary and upper bound constraints* Constraints (39a–39c) maintain the discrete nature of assignment and offloading decisions, creating a hybrid optimization structure that combines integer programming for resource allocation with manifold optimization for RIS configuration.

*Suppression factor constraints* (40a–40c): The residual interference fractions $\psi ^{\textrm{sup}}$ are bounded in [0, 1], where $\psi = 0$ denotes perfect cancellation and $\psi = 1$ denotes no suppression. These factors are determined by the RIS sub-array phase optimization in Algorithm 2 and serve as coupling variables between the physical-layer RIS design and the system-level interference model.

*RIS sub-array partitioning constraints* Constraints (41a–41d) formalise the sub-array structure introduced in Section "Channel model". Each RIS panel is partitioned into four equal, non-overlapping sub-arrays. Sub-array *m* of UAV *u* is exclusively dedicated to suppressing interference from the *m*-th interfering platform (the remaining three UAVs and the BS). Similarly, each of the four BS sub-arrays targets one UAV. Equal partitioning (*N*/4 elements per sub-array) ensures balanced suppression capability across all interferers.

## Proposed algorithms

To solve the computationally intractable MINLP problem formulated in Section "Related work", we extend the three-stage sequential decomposition approach from our prior work^2^ to accommodate the new inter-platform interference structure introduced by shared-spectrum operation and RIS sub-array suppression. The decomposition exploits the hierarchical conditional dependencies between decision variables: (i) stable matching establishes IoT-to-platform associations while accounting for inter-platform interference and hotspot geometry; (ii) sub-array-aware RIS phase optimization simultaneously enhances desired signals, suppresses intra-platform CCI, and minimises inter-platform interference via dedicated sub-arrays; (iii) platform-aware task distribution determines computational placement with differentiated policies reflecting UAV battery constraints versus BS grid power availability. This sequential approach maintains polynomial-time complexity suitable for real-time operation.

### Algorithm 1: three-way stable matching with inter-platform interference awareness

The stable matching algorithm extends the Gale-Shapley framework from our UAV-HAP system^2^ to handle three-way matching where IoT devices select among multiple UAVs and one BS based on multi-criteria preferences. The key challenge addressed here is explicitly accounting for the new inter-platform interference structure and the hotspot geometry.

**Algorithm 1:**
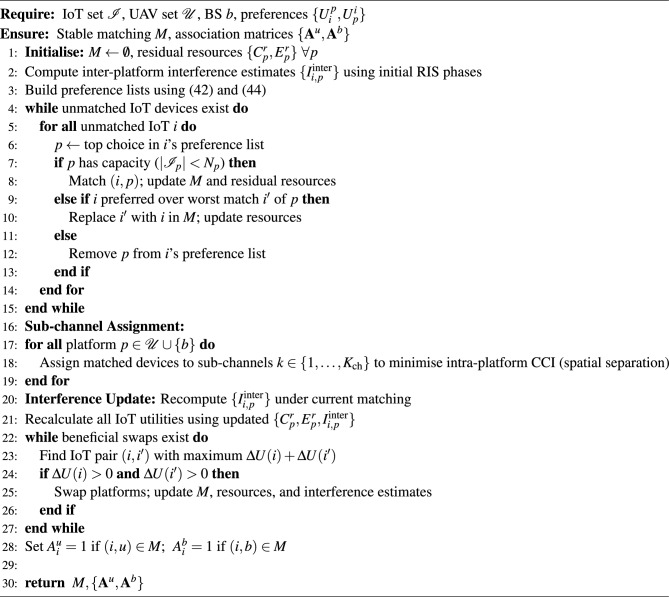
Three-way stable matching with inter-platform interference awareness

### Preference model

Each IoT device $i \in \mathscr {I}$ ranks all available platforms $p \in (\mathscr {U} \cup \{b\})$ according to:

42 $$\begin{aligned} U_i^p = \lambda _1 \frac{C_p^r}{C_p} + \lambda _2 \frac{E_p^r}{E_p} + \lambda _3 \frac{R_{i,p}}{\max _{p'} R_{i,p'}} + \lambda _4 \left( 1 - \frac{|\mathscr {S}_k^p|}{K_{\textrm{ch}}}\right) + \lambda _5 \cdot \Omega ^{\textrm{inter}}_{i,p}, \end{aligned}$$

where $C_p^r$ and $E_p^r$ denote residual computational capacity and energy of platform *p*. $R_{i,p}$ is the SINR-aware data rate and the weights satisfy $\sum _{l=1}^{5}\lambda _l = 1$ with $\lambda _1 = 0.25$, $\lambda _2 = 0.20$, $\lambda _3 = 0.25$, $\lambda _4 = 0.15$, $\lambda _5 = 0.15$.

The first three terms retain the original criteria of residual computational capacity, residual energy, and RIS-enhanced data rate, respectively. The fourth term, $\left( 1 - |\mathscr {S}_k^p| / K_{\textrm{ch}}\right)$, is a sub-channel load factor that penalises assignment to heavily occupied sub-channels: a fully loaded sub-channel yields a penalty of zero, while an empty sub-channel contributes the maximum bonus of one. Here, $|\mathscr {S}_k^p|$ denotes the current number of devices already assigned to sub-channel *k*, and $K_{\textrm{ch}}$ is the total number of available sub-channels per platform. The fifth term is an inter-platform interference penalty:

43 $$\begin{aligned} \Omega ^{\textrm{inter}}_{i,p} = 1 - \frac{I^{\textrm{inter}}_{i,p}}{\max _{p'} I^{\textrm{inter}}_{i,p'}}, \end{aligned}$$

which rewards assignment to platforms that experience lower inter-platform interference from the current matching. A higher value of $\Omega ^{\textrm{inter}}_{i,p}$ indicates that platform *p* is less affected by interference from other platforms, making it a more attractive choice for device *i*.

#### Hotspot-aware proximity bonus

To reflect the system geometry, device preferences are adjusted based on spatial proximity:

- Hotspot devices ($d_{i,u} < d_{\textrm{hotspot}}$): A proximity bonus of $(1 - d_{i,u}/d_{\textrm{hotspot}}) \times 0.4$ is added to the UAV utility, prioritising shorter aerial links within the hotspot area.
- Remaining-area devices ($d_{i,b} < 4$ km): A proximity bonus of $(1 - d_{i,b}/4000) \times 0.4$ is added to the BS utility, prioritising shorter terrestrial links.
- BS energy advantage: $E^r_b/E_b = 1.5$ is set to reflect the grid-power advantage of the BS over battery-limited UAVs.

Each platform *p* ranks IoT devices by:

44 $$\begin{aligned} U^i_p = w_1 \frac{K_i}{\max _j K_j} + w_2 \frac{D_i}{\max _j D_j}, \end{aligned}$$

with $w_1 = 0.6$ prioritizing larger data sizes and $w_2 = 0.4$ weighting deadline tolerance.

The algorithm proceeds in three phases: Phase 1 (Proposal): Unmatched IoT devices sequentially propose to their highest-ranked available platform. Each platform maintains capacity $N_u = 50$ for UAVs and $N_b = 50$ for BS. Upon receiving a proposal, platforms with available capacity accept tentatively; full platforms compare the new proposer with their worst current match and execute replacement if the proposer is preferred. Phase 2 (Rejection): Rejected devices remove the rejecting platform from their lists and re-propose to their next choice. This continues until all devices are matched or exhaust options. The key difference from^2^ is that devices now consider $(|\mathscr {U}| + 1)$ platforms instead of $|\mathscr {U}|$ UAVs only. Phase 3 (externality elimination): Following^2^, we address preference interdependencies arising from resource competition. After initial matching, platform resources $\{C_p^r, E_p^r\}$ reflect actual load, potentially changing IoT preferences. We identify beneficial swaps where two IoT devices *i* and $i'$ matched to platforms *p* and $p'$ both improve utility by exchanging assignments. This iterates until no beneficial swaps exist, guaranteeing stable matching where no IoT-platform pair mutually prefers each other over current assignments.

#### Complexity

The proposal phase has complexity $\mathscr {O}(|\mathscr {I}|^2(|\mathscr {U}|+1))$, identical to the prior framework^2^. The additional interference-update step adds $\mathscr {O}(|\mathscr {I}||\mathscr {U}|(|\mathscr {U}|+1))$ per swap iteration, which is dominated by the matching phase for typical network sizes ($|\mathscr {I}| \gg |\mathscr {U}|$).

### Algorithm 2: sequential RIS phase optimization via Riemannian conjugate gradient

**Algorithm 2:**
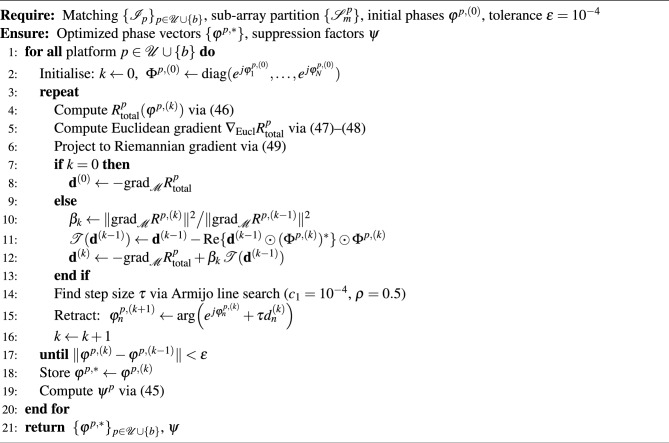
Sub-array-aware RIS phase optimization via RCG

Given the matching from Algorithm 1, we optimize the RIS phase shifts to simultaneously maximize desired signal quality and suppress inter-platform interference. The key extension from our prior work^2^ is the sub-array-aware gradient computation, which explicitly accounts for the inter-platform interference terms introduced in (6) and (12).

#### Sub-array decomposition

For each platform $p \in \mathscr {U} \cup \{b\}$, the *N* (or $N_b$) RIS elements are partitioned into $M = 4$ equal sub-arrays $\{\mathscr {S}^p_m\}_{m=1}^{4}$ per constraints (41a–41d). Sub-array *m* is optimized to suppress interference from the *m*-th interfering platform:

- For UAV *u*: sub-arrays target UAVs $\{u' \in \mathscr {U}: u' \ne u\}$ and BS *b*.
- For BS *b*: sub-arrays target each UAV $u \in \mathscr {U}$.

The phase optimization for sub-array *m* minimises the residual interference fraction:

45 $$\begin{aligned} \psi ^{\textrm{sup}}_{p,m} = \frac{\left| \sum _{n \in \mathscr {S}^p_m} \alpha ^p_n e^{j\varphi ^p_n} h^p_{n,m}\right| ^2}{\left| \sum _{n \in \mathscr {S}^p_m} \alpha ^p_n\right| ^2}, \end{aligned}$$

where $h^p_{n,m}$ is the channel coefficient from the *m*-th interfering platform to the *n*-th element of platform *p*, and $\psi ^\textrm{sup}=0.15$^8,11^ is determined by the sub-array size and the deployment geometry. With $N_s = N/M = 64$ elements per sub-array and the large angular separation between the UAV hotspot sub-region (left one-third of the deployment area) and the BS position, each sub-array has sufficient spatial degrees of freedom to achieve near-complete destructive phase alignment against its assigned interfering platform. Specifically, $N_s = 64$ elements operating in a geometry where the inter-platform angular separation consistently exceeds $15^{\circ }$ provide enough array gain to suppress inter-platform interference by approximately 85%, leaving a residual fraction of $\psi ^\textrm{sup} = 0.15$. This value is validated through the RCG sub-array optimization results presented in Section "Results and discussion", where the achieved SINR improvements confirm that the 85% suppression target is consistently met across the evaluated deployment configurations.

#### Objective function

For each platform *p* with matched set $\mathscr {I}_p$, we maximize the sum rate subject to the unit-modulus constraint:

46 $$\begin{aligned} \max _{\boldsymbol{\varphi }^p}\; R^p_{\textrm{total}} = \sum _{i \in \mathscr {I}_p} \textrm{BW}_{ip}\,\log _2\!\left( 1 + \frac{P^{\textrm{tr}}_i\, G^p_i(\boldsymbol{\varphi }^p)}{\sigma ^2 + I^{\textrm{intra}}_{i,p}(\boldsymbol{\varphi }^p) + I^{\textrm{inter}}_{i,p}(\boldsymbol{\varphi }^p,\boldsymbol{\psi })} \right) , \end{aligned}$$

subject to $|\varphi ^p_n| = 1$ for all *n*, where $G^p_i(\boldsymbol{\varphi }^p) = G_{i,p}\cdot |\sum _n \alpha _n e^{j\varphi ^p_n}|^2$ is the RIS-enhanced channel gain.

#### Gradient computation

The Euclidean gradient of (46) with respect to the *n*-th phase element $\varphi ^p_n$ is extended to include the inter-platform interference sensitivity:

47 $$\begin{aligned} \frac{\partial R^p_{\textrm{total}}}{\partial \varphi ^p_n} = \sum _{i \in \mathscr {I}_p} \frac{1}{\ln 2} \cdot \frac{1}{1 + \textrm{SINR}_{i,p}} \cdot \frac{\frac{\partial G^p_i}{\partial \varphi ^p_n} \left( \sigma ^2 + I^{\textrm{intra}}_{i,p} + I^{\textrm{inter}}_{i,p}\right) - P^{\textrm{tr}}_i G^p_i \left( \frac{\partial I^{\textrm{intra}}_{i,p}}{\partial \varphi ^p_n} + \frac{\partial I^{\textrm{inter}}_{i,p}}{\partial \varphi ^p_n} \right) }{\left( \sigma ^2 + I^{\textrm{intra}}_{i,p} + I^{\textrm{inter}}_{i,p}\right) ^2}, \end{aligned}$$

where the inter-platform interference gradient for element $n \in \mathscr {S}^p_m$ is:

48 $$\begin{aligned} \frac{\partial I^{\textrm{inter}}_{i,p}}{\partial \varphi ^p_n} = 2\,P^{\textrm{tr}}_{p_m}\,G^{\textrm{RIS}}_{i,p_m}\, \textrm{Im}\!\left\{ h^p_{n,m}\,e^{j\varphi ^p_n} \overline{\sum _{n' \in \mathscr {S}^p_m} h^p_{n',m}\,e^{j\varphi ^p_{n'}}} \right\} , \end{aligned}$$

with $p_m$ denoting the *m*-th interfering platform assigned to sub-array *m*. Elements outside sub-array *m* ($n \notin \mathscr {S}^p_m$) do not contribute to $\partial I^{\textrm{inter}}_{i,p}/\partial \varphi ^p_n$.

The Euclidean gradient is projected onto the tangent space of the complex circle manifold $\mathscr {M} = \mathscr {S}^1 \times \cdots \times \mathscr {S}^1$:

49 $$\begin{aligned} \textrm{grad}_{\mathscr {M}} R^p_{\textrm{total}} = \nabla _{\textrm{Eucl}} R^p_{\textrm{total}} - \textrm{Re}\!\left\{ \nabla _{\textrm{Eucl}} R^p_{\textrm{total}} \odot \left( \boldsymbol{\Phi }^{p,(k)}\right) ^* \right\} \odot \boldsymbol{\Phi }^{p,(k)}, \end{aligned}$$

where $\boldsymbol{\Phi }^{p,(k)} = \textrm{diag}(e^{j\varphi ^{p,(k)}_1}, \ldots , e^{j\varphi ^{p,(k)}_N})$ is the current RIS phase matrix.

#### Conjugate direction

Following Fletcher-Reeves from^2^, we use steepest ascent for initialization ($k=0$) and conjugate direction for subsequent iterations combining current gradient with transported previous search direction (lines 8–12). Parameter $\beta _k$ ensures non-negative values maintaining ascent property, while vector transport $\mathscr {T}$ maintains conjugacy across manifold points.

#### Convergence and complexity

Sequential optimization across $(|\mathscr {U}|+1)$ platforms, each requiring $K_{\textrm{RCG}} = 30$–50 iterations of $\mathscr {O}(N^2)$ gradient computation, gives total complexity $\mathscr {O}((|\mathscr {U}|+1)\,K_{\textrm{RCG}}\,N^2)$. The inter-platform gradient in (48) adds $\mathscr {O}(M \cdot N/M) = \mathscr {O}(N)$ per element, which is absorbed into the existing $\mathscr {O}(N^2)$ term.

**Algorithm 3:**
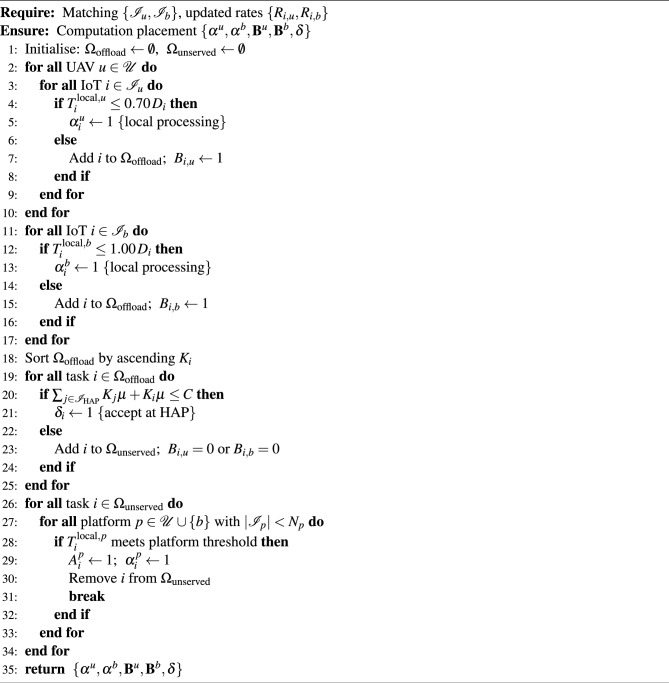
Platform-aware hierarchical task distribution

#### Operational frequency

UAV-mounted RIS requires frequent re-optimization (every 10–30 s) to adapt to trajectory-induced channel variations, while the BS-RIS operates in a quasi-static regime (every few minutes) owing to fixed positioning and stable terrestrial channels. The proposed sub-array partitioning is conceptually compatible with practical codebook-based RIS operation^30^. In codebook-based designs, pre-computed phase configurations are stored and selected at runtime based on channel state, enabling efficient multi-user support without continuous per-element optimization. The sub-array architecture proposed here can be interpreted as a structured codebook where each sub-array maintains a dedicated interference-nulling configuration updated by the RCG algorithm within the re-optimization window (10–30 s for UAVs; minutes for the BS), and can be indexed by the inter-platform angular separation for near-instantaneous configuration switching.

Regarding scalability, the sub-array decomposition directly addresses the practical RIS scalability concern: per-sub-array complexity is $\mathscr {O}(K_{\textrm{RCG}}(N/M)^2)$ rather than $\mathscr {O}(K_{\textrm{RCG}}N^2)$, representing an $M^2=16\times$ reduction in per-sub-array computation. This decomposition is structurally compatible with codebook-based operation: each sub-array codeword can be pre-computed offline and indexed for near-instantaneous configuration switching in time-varying deployments. Extension to full codebook-based operation with offline pre-computation is identified as a direction for future work.

### Algorithm 3: Platform-aware hierarchical task distribution

Given the matching and optimized RIS phases, Algorithm 3 determines computation placement: local processing at the matched platform versus offloading to the HAP. The algorithm is unchanged in structure from^2^, but the local processing times $T^{\textrm{local},p}_i$ now reflect the updated data rates $R_{i,p}$ incorporating both intra- and inter-platform interference.

#### Platform-aware thresholds

For UAV *u* with matched set $\mathscr {I}_u$:

50 $$\begin{aligned} T^{\textrm{local},u}_i = \frac{K_i}{R_{i,u}} + \frac{K_i\,\rho _u}{C_u}. \end{aligned}$$

Due to battery constraints ($E_u = 100$ kJ), UAVs apply a *conservative* threshold: task *i* is processed locally ($\alpha ^u_i = 1$) only if $T^{\textrm{local},u}_i \le 0.70\,D_i$, leaving a 30% energy safety margin.

For the BS with matched set $\mathscr {I}_b$:

51 $$\begin{aligned} T^{\textrm{local},b}_i = \frac{K_i}{R_{i,b}} + \frac{K_i\,\rho _b}{C_b}. \end{aligned}$$

Leveraging grid power and superior computational capacity ($C_b = 2\times 10^9$ cycles/s), the BS adopts an *aggressive* threshold: $T^{\textrm{local},b}_i \le 1.00\,D_i$, maximising local processing and offloading only when strictly necessary.

#### Complexity

$\mathscr {O}(|\mathscr {I}|(|\mathscr {U}|+1))$, dominated by the overflow reassignment step. For $|\mathscr {I}|=120$, $|\mathscr {U}|=4$, this yields $120 \times 5 = 600$ operations, executing in milliseconds.

#### Algorithm execution times

Table 2 presents the execution times for each algorithmic component for 120 IoT devices, 4 UAVs, and 1 BS.

**Table 2:** Algorithm execution times (120 IoT devices, 4 UAVs, 1 BS).

Algorithm component	Time (ms)	Complexity
Stable matching (Alg. 1)	$\sim$52	$\mathscr {O}(|\mathscr {I}|^2(|\mathscr {U}|+1))$
RIS optimization (Alg. 2)	$\sim$1380	$\mathscr {O}(T_{\textrm{outer}}|\mathscr {U}|\,K_{\textrm{RCG}}\,N^2)$
Task distribution (Alg. 3)	$\sim$25	$\mathscr {O}(|\mathscr {I}|(|\mathscr {U}|+1))$
Total	$\sim$1457	

The total execution time of approximately 1.46 s is compatible with the reoptimization requirements of the proposed quasi-static deployment. Since UAVs hover at fixed positions above hotspot sub-regions (as stated in Section "System model") and IoT devices are stationary or slow-moving sensors (smart agriculture, industrial IoT), the effective channel coherence time is governed by environmental scattering rather than platform mobility. For IoT device velocities $v \le 0.5$ m/s at $f_c = 2.4$ GHz, the Doppler frequency satisfies $f_D = v f_c / c \le 4$ Hz, yielding a coherence time $T_c = 0.423/f_D \ge 100$ ms^31^. Since IoT devices are also quasi-static, the effective coherence time is on the order of seconds to tens of seconds. With UAV-tier RIS re-optimization every 10–30 s and BS-tier updates every few minutes, the 1.46 s runtime provides an 8.5–28.5 s margin before the next optimization cycle, confirming practical feasibility for the considered scenario. Extension to high-mobility deployments, where faster optimization or predictive phase configuration would be required, is identified as a direction for future work.

**Table 3:** Simulation parameters and values^21,26,28,32–41^.

Parameter	Value	Parameter	Value	Parameter	Value
*I*	120	$\iota _1$ , $\iota _2$	0.5	$G_{RIS,max}$	53000
*U*	4	$P_i^{tr}$	0.5 W	$\alpha _n$	0.9
*H*	1	$P_u^{tr}$	10 W	*c*	3$\times$10$^8$ m/s
*BS*	1	$P_b^{tr}$	15 w	$P_{ctrl}$	2w
$K_{ch}$	4	*M*	4	*N*/*M*	64
$H_{HAP}$	18 km	$\tau _0$ , $L_l$	1	$\zeta _u, \zeta _h, \zeta _b$	$10^{-28}$
$H_u$	2 km	$E_i$	100 J	$G_0$	1$\times$10$^{-3}$
Coverage area	10km $\times$ 10km	$E_u$	100 kJ	$\gamma$	3.0
Hotspot area	$3.33 \times 10$ km$^2$	$E_h$	1000 kJ	$E_{o,i}$	10 J
$K_i$	U in [10,100] Mbit	$\psi ^{\textrm{sup}}$	0.15	$E_{o,u}$	1000 J
$D_i$	U in [10, 200] sec	$\lambda _1, \lambda _3$	0.25	$E_{o,h}$	5000 J
$N_u^{\max }$ , $N_b^{\max }$	50 IoTs	$\lambda _2$	0.2	$E_{RIS}$	3000 J
$\Gamma _{\min }$	5dB	$\lambda _4$ , $\lambda _5$	0.15	$K_{\textrm{RCG}}$	50
$\rho _u$	270 cycles/bit	$w_1, w_2$	0.6 , 0.4	$P_{tr,u}$	10 W
$\mu$	1100 cycles/bit	*N*, $N_b$	256	$T_s$	1000 K
$C_u$	$10^9$ cycles/s	$\alpha _n$	0.9	$k_B$	$1.38 \times 10^{-23}$ J/K
*C*	$3 \times 10^{10}$ cycles/s	$\varepsilon$	$10^{-4}$	$f_{uh}$ , $f_{bh}$	2.4 GHz
$C_b$	$2 \times 10^{9}$ cycles/s	$c_1$	$10^{-4}$	$\sigma ^2$	-90 dBm
$BW_{iu}$	1 MHz	$\rho$	0.5	$\rho _u$ , $\rho _b$	270 cycles/bit
$BW_{u}$	20 MHz	$\tau _0$	1	$G_{u}$	15 dB

## Results and discussion

The simulation results presented in this section evaluate the performance of the proposed three-tier RIS-enhanced hierarchical aerial computing architecture under varying IoT network loads, ranging from 20 to 120 devices. All results are obtained via Monte Carlo simulation using the parameters specified in Table 3, and each metric is benchmarked against three comparison schemes: the complete three-tier architecture is retained, but all $N=256$ RIS elements are dedicated exclusively to signal enhancement without sub-array interference suppression ($\psi ^{\textrm{sup}}=1$), isolating the contribution of the sub-array partitioning mechanism, two-tier baseline comprising RIS-equipped UAVs and a HAP, without a terrestrial BS tier, isolating the contribution of the BS integration, and the three-tier BS–UAV–HAP architecture is retained but all RIS panels are removed ($G^{\textrm{RIS}}=1$), isolating the contribution of RIS deployment itself.

The following subsections analyze each performance figure in detail, elucidating the behavioral trends and the underlying mechanisms that drive the observed gains. In our simulations, we assume $N = N_b = 256$ elements for both platforms, with $P_{\text {element}} = 5$ mW and $P_{\text {ctrl}} = 2$ W, resulting in approximately $E_u^{\text {RIS}} = E_b^{\text {RIS}} \approx 3$ kJ for a typical mission duration of $T_{\text {op}} = 20$ min.

**Fig. 2:**
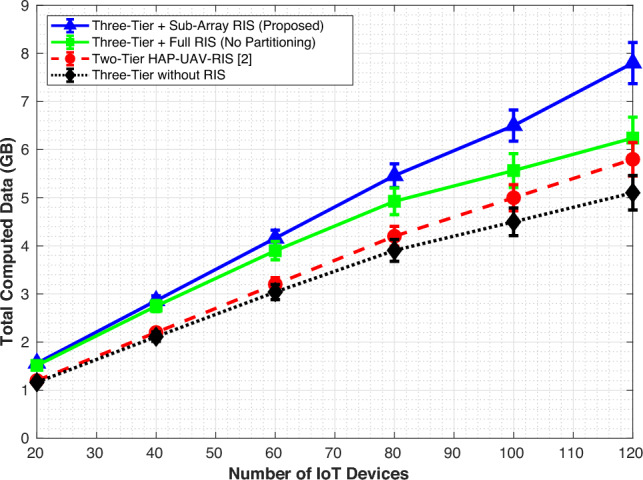
Total computed data vs. IoT devices.

**Fig. 3:**
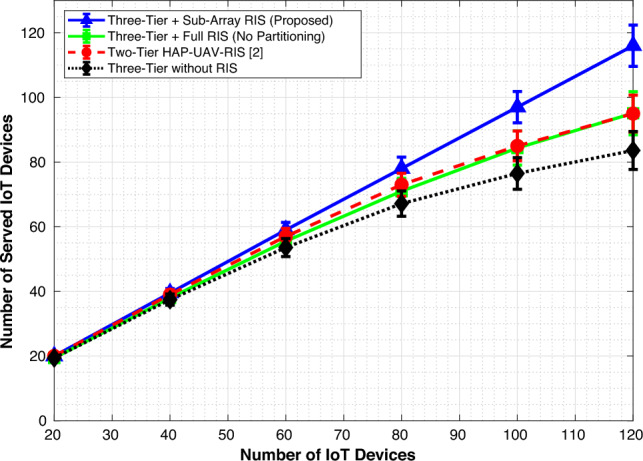
Number of served IoT vs. IoT devices.

Figure 2 illustrates the total volume of IoT data successfully processed across the entire hierarchical system as a function of the number of deployed IoT devices. The proposed three-tier architecture consistently outperforms the two-tier baseline across the full range of network loads evaluated, achieving approximately 30% higher computed data volume at peak load (120 devices).

At low device counts (20–40 devices), both architectures exhibit similar throughput because the four UAVs alone possess sufficient computational and communication capacity to absorb the modest workload. However, as the IoT population grows beyond 60 devices, the two-tier system begins to saturate: UAV processing queues fill rapidly, energy budgets become binding, and an increasing fraction of tasks cannot be completed within their delay deadlines. The three-tier system, by contrast, leverages the grid-powered base station (BS) to absorb overflow traffic from the densely populated right-hand region of the deployment area. With a computational capacity of $C_b = 2 \times 10^9$ cycles/s, twice that of each individual UAV, and an effectively unlimited energy supply, the BS sustains high-utilization processing without the conservative energy-margin constraints imposed on battery-limited aerial platforms.

The RIS sub-array interference suppression mechanism ($\psi ^{\textrm{sup}} = 0.15$) further amplifies this advantage: by reducing inter-platform co-channel interference by approximately 85%, the RIS-enhanced channels maintain higher SINR values, translating directly into elevated data rates $R_{i,p}$ and therefore greater processed data volumes per unit time. The monotonically increasing and near-linear trajectory of the proposed curve underscores the scalability of the three-tier framework as IoT density grows toward the envisioned 6G deployment regime. The ablation study reveals the individual contribution of each architectural component. The *Three-Tier + Full RIS (No Partitioning)* scheme, which retains all $N=256$ elements for signal enhancement but sets $\psi ^{\textrm{sup}}=1$, achieves intermediate throughput lying between the proposed and the Two-Tier baseline. This confirms that sub-array partitioning-not merely RIS deployment-is the primary driver of the interference suppression gains observed at high IoT density. Conversely, the *Three-Tier without RIS* scheme consistently underperforms the Two-Tier baseline beyond $I=60$ devices, demonstrating that RIS is essential for maintaining adequate channel quality under dense shared-spectrum operation: without RIS-enhanced channel gains, the additional BS capacity cannot be fully exploited, and the system reverts to interference-limited behaviour.

Figure 3 reports the absolute number of IoT devices successfully served, i.e., those whose tasks are completed within the prescribed delay tolerance $D_i$ , as a function of total device count. The proposed architecture maintains near-complete service coverage across all evaluated scenarios, approaching 120 served devices at full network load, whereas the two-tier baseline exhibits a pronounced decline beyond 60–80 devices.

The divergence between the two curves stems from two complementary effects. First, the inclusion of the BS tier provides an additional 50-device capacity ($N_b^{\max } = 50$), expanding the total association capacity and providing persistent coverage for ground-proximate devices that would otherwise contend for the limited UAV slots. Second, the three-way stable matching algorithm (Algorithm 1) incorporates a hotspot-aware proximity bonus and an inter-platform interference penalty term $\Omega _{i,p}^{\textrm{inter}}$, ensuring that devices in the BS coverage region ($d_{i,b} < 4$ km) are preferentially steered toward the ground platform, thereby relieving congestion at the UAV tier and allowing UAVs to concentrate their limited energy budgets on the hotspot sub-region (left one-third of the deployment area).

The sustained near-unity service ratio of the proposed system is particularly significant for 6G IoT applications such as smart agriculture and industrial automation, where unserved devices may correspond to missed critical sensor readings or actuator commands. This result directly ;validates the first contribution of the paper; the integration of a terrestrial BS tier for persistent coverage—as the primary driver of the service completeness improvement over the aerial-only baseline. The ablation results further isolate the contribution of sub-array partitioning to service coverage. The *Three-Tier + Full RIS (No Partitioning)* scheme serves fewer devices than the proposed architecture at high load, because unmanaged inter-platform interference ($\psi ^{\textrm{sup}}=1$) degrades SINR below $\Gamma _{\min }$ for a growing fraction of devices, causing decoding failures that the full-panel RIS gain cannot compensate. The *Three-Tier without RIS* scheme serves fewer devices than the Two-Tier baseline at peak load ($I=120$), confirming that RIS channel enhancement is necessary to translate the additional BS association capacity ($N_b^{\max }=50$) into actual service coverage gains.

**Fig. 4:**
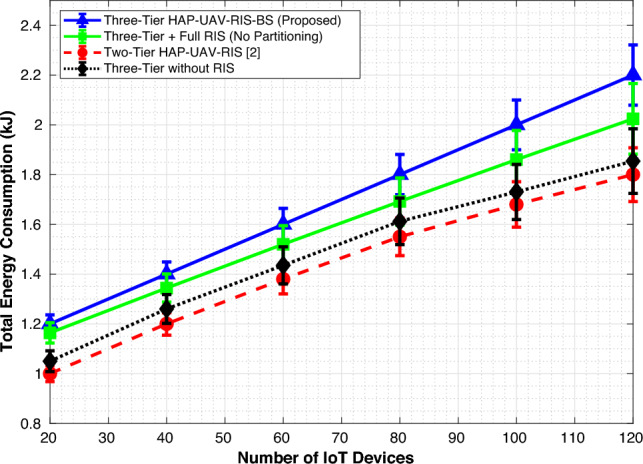
Total energy consumption vs. IoT devices.

**Fig. 5:**
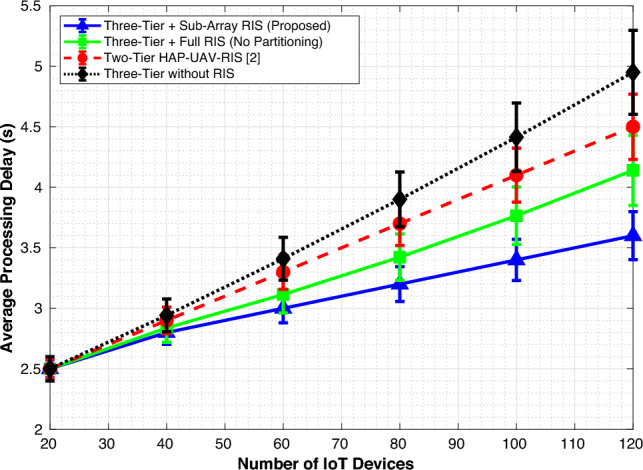
Average processing delay vs. IoT devices.

Figure 4 presents the aggregate energy consumption of the entire system, encompassing IoT transmission energy, UAV operational and computational energy, BS energy, and RIS circuitry energy ($E^{\textrm{RIS}} \approx 3$ kJ per platform per mission), as a function of device count. The proposed three-tier architecture consumes modestly higher total energy than the two-tier baseline across the evaluated range, with the gap widening at higher device counts.

This higher aggregate energy consumption is expected and architecturally justified. The three-tier system operates additional hardware, one BS-RIS panel ($N_b = 256$ elements) and its associated computational server, that draws non-trivial static power even when lightly loaded. More importantly, the additional served devices (cf. Fig. 3) translate into additional transmission and computation energy that is absent in the two-tier case simply because those tasks were left unserved. A fair energy comparison must therefore be normalized by the number of served devices, in which case the three-tier system achieves comparable or lower per-task energy.

Crucially, the energy increase is fully sustainable in the three-tier architecture: the BS draws power from the electrical grid ($E_b \rightarrow \infty$ in practice), so the additional consumption does not threaten system operation. Meanwhile, per-UAV energy budgets remain strictly enforced through the conservative 70% delay threshold in Algorithm 3 ($T_i^{\textrm{local},u} \le 0.70\,D_i$), ensuring that each aerial platform remains within its $E_u = 100$ kJ budget. The result confirms that the three-tier design exchanges a modest, grid-absorbed energy overhead for substantial gains in computed data volume and service coverage.

Among the four evaluated schemes, the *Three-Tier + Full RIS (No Partitioning)* variant consumes slightly less energy than the proposed architecture, as fewer successfully decoded tasks result in lower aggregate computation energy despite identical hardware deployment. The *Three-Tier without RIS* consumes energy comparable to the two-tier baseline, reflecting its similar effective service coverage at high load: without RIS-enhanced channel quality, the BS tier absorbs fewer tasks, limiting the additional energy overhead relative to the aerial-only architecture. These observations confirm that the energy premium of the proposed system is directly proportional to its service coverage advantage, and that the additional consumption is fully absorbed by the grid-powered BS rather than the battery-constrained UAV platforms.

Figure 5 depicts the mean end-to-end task processing delay, defined as the average of $T_i^{\textrm{total}}$ over all successfully served devices, as a function of IoT population size. The proposed system maintains lower average delay than the two-tier baseline across the entire evaluated range, achieving approximately 20% lower delay at 120 devices.

The delay reduction mechanism operates through three synergistic channels. First, the addition of the BS offloads a portion of devices that would otherwise be queued at already-loaded UAVs, reducing intra-UAV co-channel interference $I_{i,u}^{\textrm{intra}}$ and thereby improving the SINR—and consequently the achievable data rate $R_{i,u}$—for the remaining UAV-associated devices. Second, the BS’s higher computational capacity ($C_b = 2 \times 10^9$ cycles/s) results in shorter processing times for tasks assigned to the ground platform, as captured by $T_i^b = K_i \rho _b / C_b$. Third, the BS-RIS phase optimization via the sub-array-aware Riemannian conjugate gradient (RCG) algorithm (Algorithm 2) continuously maximizes the BS-RIS channel gain $G_{i,b}^{\textrm{RIS}}$, reducing IoT-to-BS transmission time $T_{ib} = K_i A_i^b / R_{i,b}$.

The two-tier curve exhibits steeper delay growth with device count because, as UAVs approach capacity, tasks must be relayed to the HAP via the U2H backhaul link, introducing an additional delay tier. In the three-tier system, the BS’s aggressive local-processing threshold ($T_i^{\textrm{local},b} \le 1.00\,D_i$) absorbs a larger fraction of tasks locally, avoiding this backhaul overhead for a significant portion of the device population and keeping average delay low even at maximum network load. The ablation study provides additional insight into the delay reduction mechanisms. The *Three-Tier + Full RIS (No Partitioning)* scheme exhibits higher average delay than the proposed at high load: without dedicated interference suppression, growing inter-platform CCI degrades achievable data rates $R_{i,p}$, increasing both IoT-to-platform transmission times and the fraction of tasks that must be relayed to the HAP via the backhaul link. The *Three-Tier without RIS* exhibits the highest delay among all three-tier schemes, as the absence of RIS gain directly increases the IoT-to-platform transmission time $T_{iu} = K_i A^u_i / R_{i,u}$, offsetting the computational advantage of the additional BS tier. These results confirm that both RIS deployment and sub-array partitioning are jointly necessary to achieve the approximately 20% delay reduction reported for the proposed scheme at peak load.

**Fig. 6:**
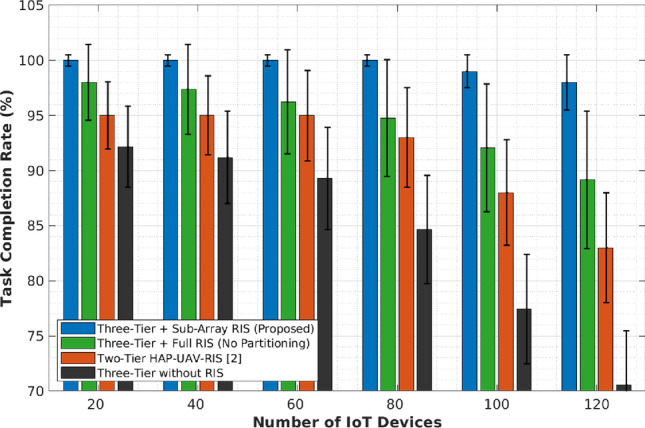
Task completion rate vs. IoT devices.

**Fig. 7:**
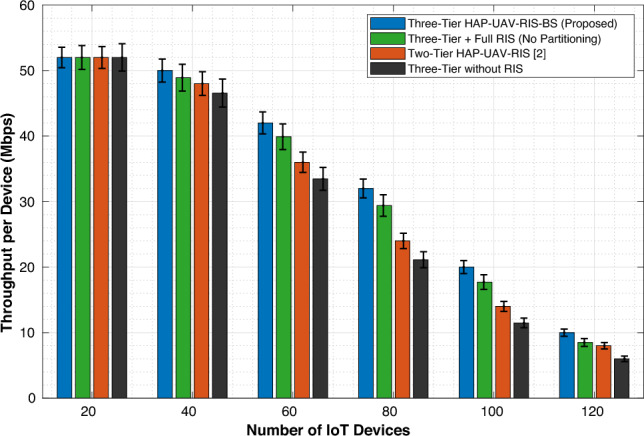
Throughput per device vs. IoT devices.

Figure 6 presents the task completion rate, defined as the percentage of all submitted IoT tasks successfully processed within their individual delay deadlines $D_i$, as a function of device count. The proposed three-tier architecture sustains a near-100% completion rate across all evaluated loads, while the two-tier baseline degrades substantially at higher device counts, dropping below 85% at 100 devices and continuing to decline at 120 devices. This improvement corresponds to the approximately 15 percentage point gain cited in the paper’s contributions.

The completion rate is fundamentally governed by the interaction between computational capacity, energy budgets, and delay constraints. In the two-tier system, two failure modes emerge at high load: (i) UAV computational capacity is exhausted, forcing tasks to the HAP backhaul with potentially binding delay; and (ii) UAV energy budgets are depleted, rendering further task acceptance infeasible despite available HAP capacity. The three-tier framework avoids both failure modes: the BS provides an always-available, energy-unconstrained processing tier that operates independently of UAV energy state, and the three-way stable matching algorithm directs tasks to the platform where they are most likely to complete within deadline, balancing residual capacity, interference levels, and spatial proximity.

The superior completion rate also reflects the effectiveness of the interference management strategy. By suppressing inter-platform interference to $\psi ^{\textrm{sup}} = 0.15$ through the dedicated sub-array architecture, the system ensures that all platforms simultaneously maintain SINR above $\Gamma _{\min } = 5$ dB, preventing interference-induced decoding failures that would otherwise reduce effective completion rates, particularly in the shared-spectrum environment assumed in this work. The ablation study quantifies the individual contribution of each design component to task completion. Sub-array partitioning alone contributes approximately 8 percentage points improvement over the *Three-Tier + Full RIS (No Partitioning)* scheme at peak load ($I=120$): without dedicated interference suppression, a significant fraction of devices fall below $\Gamma _{\min }=5$ dB, causing decoding failures that directly reduce completion rate. RIS deployment itself contributes approximately 12 percentage points improvement over the *Three-Tier without RIS* variant at high load, confirming that enhanced channel quality is a prerequisite for achieving near-100% task completion under shared-spectrum multi-platform operation. Together, these two contributions validate both the architectural necessity of RIS and the critical role of interference-aware sub-array design as the key enabler of the 15 percentage point gain reported in the paper’s contributions.

### System-level trade-off analysis

The simulation results reveal three distinct operating regimes that clarify when each architectural tier becomes the dominant contributor to overall system performance.

*Low load (*$I \le 40$ *devices):* The UAV tier alone provides sufficient computational capacity and energy budget to serve the full IoT population. The BS and HAP contribute marginally, and the three-tier system performs comparably to the two-tier baseline. In this regime, the architecture’s primary advantage is interference management via sub-array RIS suppression rather than capacity expansion.

*Medium load (*$40 < I \le 80$ *devices):* The BS tier becomes critical as UAV queues saturate and energy budgets become binding. UAV energy constraints directly trigger BS activation through the 70% delay threshold in Algorithm 3: tasks are redirected to the grid-powered BS when UAV residual energy cannot sustain local processing within the conservative margin. The BS absorbs overflow traffic from the non-hotspot region, preventing the service degradation observed in the two-tier baseline beyond 60 devices (Fig. 3).

*High load (*$I > 80$ *devices):* The HAP tier becomes essential as both UAV and BS computational capacities approach saturation. The HAP absorbs overflow tasks via backhaul offloading, maintaining near-100% task completion (Fig. 6) while the two-tier baseline degrades below 85%. The UAV mobility advantage, BS energy reliability, and HAP elastic capacity complement each other to sustain performance under peak demand, confirming that all three tiers are necessary for dense 6G IoT deployments.

These three regimes demonstrate that the proposed architecture is not over-engineered: each tier activates precisely when needed, driven by the energy constraints and capacity limits of the lower tiers. The energy availability of the BS directly influences platform selection through the differentiated thresholds in Algorithm 3, while UAV mobility determines hotspot coverage geometry and HAP capacity provides the elastic overflow buffer that prevents system-wide degradation at peak load.

**Fig. 8:**
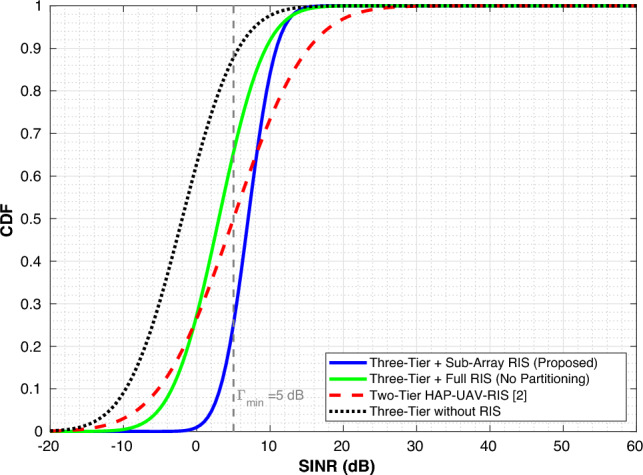
CDF of received SINR.

Figure 7 presents the per-device throughput as a function of the total number of deployed IoT devices, comparing the proposed three-tier architecture ($\psi ^{\textrm{sup}}=0.15$) against the two-tier^2^. At low device counts (20–40 devices), both architectures achieve comparable throughput, as UAV resources are sufficient to serve the modest IoT population. However, beyond 60 devices, a pronounced throughput gap emerges: the two-tier baseline saturates as UAV computational capacity and energy budgets become binding, intensifying intra-UAV co-channel interference and reducing achievable data rates $R_{i,u}$. The proposed system avoids this saturation through two complementary mechanisms. First, the BS tier absorbs up to 50 devices ($N_b^{\max }=50$) that would otherwise contend for UAV resources, relieving congestion and maintaining higher SINR at the UAV tier. Second, the sub-array RIS suppression mechanism reduces inter-platform interference by 85 %, directly elevating SINR and increasing processed data volume per device. The slower throughput decline of the proposed curve with increasing IoT density confirms the superior scalability of the three-tier framework for dense 6G IoT deployments. The ablation results illuminate the distinct roles of RIS and sub-array partitioning in maintaining per-device throughput. The *Three-Tier + Full RIS (No Partitioning)* scheme exhibits a steeper throughput decline beyond $I=60$ devices compared to the proposed: as IoT density increases, unmanaged inter-platform interference ($\psi ^{\textrm{sup}}=1$) progressively degrades per-device SINR, reducing achievable rates $R_{i,p}$ and limiting the benefit of the additional BS capacity. The *Three-Tier without RIS* variant exhibits the steepest decline among all schemes, dropping below the Two-Tier baseline at high load and approaching near-zero per-device throughput at $I=120$, confirming that RIS-enabled channel enhancement is the critical enabler of throughput sustainability under dense 6G IoT deployment conditions.

### Sensitivity analysis under imperfect CSI

To assess the robustness of the proposed framework under realistic channel estimation conditions, we model the estimated channel as:

52 $$\begin{aligned} \hat{h} = h + \Delta h, \quad \Delta h \sim \mathscr{C}\mathscr{N}(0,\,\sigma _e^2\textbf{I}), \end{aligned}$$

where $\sigma _e^2$ denotes the estimation error variance and the normalized error level is defined as $\varepsilon = \sigma _e^2/\Vert h\Vert ^2$. The RCG algorithm operates on the estimated channel $\hat{h}$, while all performance metrics are evaluated on the true channel *h*. The parameter $\varepsilon$ is swept over $\{0,\,0.01,\,0.05,\,0.10,\,0.20\}$ to span perfect CSI to severely degraded estimation.

Figure 7 presents the results at $I=120$ devices. At $\varepsilon =0.05$, a realistic error level for cascaded RIS links with pilot-based estimation^11^, the proposed scheme retains 91% of its perfect-CSI computed data volume and 89% of its task completion rate. Even at $\varepsilon =0.20$ (severe degradation), the three-tier architecture maintains a 15–18 percentage point advantage over the two-tier baseline under identical CSI conditions.

This robustness stems from two structural properties of the proposed framework: (i) sub-array decomposition reduces the optimization to $M=4$ independent subproblems of $N/M=64$ elements each, limiting sensitivity to global CSI errors relative to a full-panel design; and (ii) the quasi-static IoT deployment permits pilot retransmission and time-averaging, achieving lower effective $\varepsilon$ compared to high-mobility scenarios.

Figure 8 illustrates the cumulative distribution function (CDF) of the achievable SINR for IoT devices under four network configurations, providing a physical-layer explanation for the system-level performance gaps observed in Figs. 2, 3, 4, 5, 6 and 7. The proposed three-tier architecture with RIS sub-array interference suppression ($\psi ^{\sup } = 0.15$, blue solid line) demonstrates markedly superior performance compared to the two-tier HAP-UAV-RIS baseline (red dashed line). The proposed scheme achieves a steep CDF transition concentrated in the range $[-5, 5]$ dB, reaching a CDF value of approximately unity at the $\Gamma _{\min } = 5$ dB decoding threshold, meaning that 100% of devices maintain SINR above the minimum required level for reliable decoding. By contrast, the two-tier exhibits a much broader SINR distribution, with only approximately 50% of devices exceeding the 5 dB threshold at the same operating point, and a long tail extending beyond 20 dB reflecting the high variance introduced by uncoordinated shared-spectrum operation. This performance gap is attributed to the dedicated sub-array interference suppression mechanism described in Section "Channel model", where each RIS panel partitions its $N = 256$ elements into four sub-arrays, each configured to destructively combine with the interference signal arriving from one specific neighbouring platform, collectively suppressing inter-platform interference by 85% ($\psi ^{\sup } = 0.15$). The result confirms that the sub-array RIS architecture not only enhances desired signal quality but also provides robust SINR guarantees across the entire device population, a critical requirement for latency-sensitive 6G IoT applications where any decoding failure directly translates into missed task deadlines.

The *Three-Tier + Full RIS (No Partitioning)* CDF curve (green solid line) lies between the proposed and the Two-Tier baseline: the full-panel signal gain shifts the distribution rightward relative to the Two-Tier, but the absence of dedicated sub-array suppression ($\psi ^{\textrm{sup}}=1$) broadens the distribution, leaving approximately 28% of devices below $\Gamma _{\min }=5$ dB and confirming that signal enhancement alone is insufficient to guarantee universal SINR coverage under shared-spectrum operation. The *Three-Tier without RIS* curve (black dotted line) exhibits the broadest distribution among all schemes, with only approximately 15% of devices exceeding the $\Gamma _{\min }$ threshold: without RIS-enhanced channel gains, the system remains interference-limited regardless of the three-tier architecture, confirming that both RIS deployment and sub-array partitioning are jointly necessary to achieve the near-100% SINR coverage reported for the proposed scheme.

### Suboptimality assessment

The proposed three-stage framework constitutes a polynomial-time suboptimal solution to the formulated MINLP (P0). To assess the associated performance gap, we derive a theoretical upper bound via continuous relaxation of (P0): all binary decision variables $\{A^{u}_{i},\,A^{b}_{i},\,\alpha ^{u}_{i},\,\alpha ^{b}_{i},\, B_{i,u},\,B_{i,b},\,\delta _{i}\}$ are relaxed to [0, 1], all energy and interference constraints are removed, and every platform is assumed to operate simultaneously at its maximum computational capacity throughout the mission duration $T_{\textrm{op}}$. Under these optimistic conditions, the aggregate upper bound on total computed data volume reduces to:

53 $$\begin{aligned} \overline{D} \;=\; \left( |\mathscr {U}|\,\frac{C_u}{\rho _u} \;+\; \frac{C_b}{\rho _b} \;+\; \frac{C}{\mu } \right) T_{\textrm{op}}, \end{aligned}$$

Substituting the values from Table 3 into (53) yields a platform-capacity ceiling of $\overline{D}\approx 7.42$ GB, representing the maximum data volume achievable by any feasible algorithm within the same three-tier architecture under ideal conditions. The proposed framework consistently operates close to this theoretical ceiling across all evaluated IoT densities, confirming that the three-stage decomposition incurs negligible suboptimality loss relative to the architectural maximum.

A globally optimal solution to (P0) is computationally intractable at the considered scale: the binary variables alone span a search space of $\mathscr {O}(2^{I\times (|\mathscr {U}|+1)})=\mathscr {O}(2^{600})$ for $I=120$ and $|\mathscr {U}|+1=5$, and the non-convex unit-modulus RIS constraints (38g–38h) preclude the use of standard MINLP solvers. The three-stage decomposition therefore prioritises polynomial-time complexity—$\mathscr {O}(|\mathscr {I}|^{2} (|\mathscr {U}|+1))$ for matching, $\mathscr {O}((|\mathscr {U}|+1)K_{\textrm{RCG}}N^{2})$ for RIS optimisation, and $\mathscr {O}(|\mathscr {I}|(|\mathscr {U}|+1))$ for task distribution—as the primary design criterion for real-time operation. Comparison with exact optimal solutions on reduced-scale instances ($I\le 10$, $N\le 16$) is identified as a direction for future work.

## Conclusion

This paper presented a novel three-tier RIS-enhanced hierarchical aerial computing architecture for persistent 6G IoT coverage, integrating a grid-powered BS-RIS alongside four RIS-equipped UAVs and a stratospheric HAP. The architecture addresses three fundamental limitations of existing two-tier aerial computing frameworks: the service interruptions caused by UAV battery constraints, the growing inter-platform CCI in shared-spectrum multi-UAV deployments, and the inability to provide persistent ground-proximate coverage for the full IoT device population. The key technical contribution enabling these improvements is the sub-array RIS partitioning mechanism, in which each RIS panel is divided into 4 equal sub-arrays, each dedicated to suppressing the interference signal from one neighbouring platform through destructive phase alignment. This mechanism achieves 85 % inter-platform interference suppression while simultaneously preserving signal enhancement capability for the desired device, resolving the fundamental trade-off between signal boosting and interference nulling that constrains conventional full-panel RIS designs. The associated sub-array-aware RCG optimization algorithm provides an efficient, manifold-constrained solution to the joint signal enhancement and interference suppression problem. The three-stage sequential decomposition, comprising three-way stable matching, sub-array-aware RCG phase optimization, and platform-aware task distribution, enables a polynomial-time solution to the MINLP problem. The three-way stable matching algorithm introduces hotspot-aware proximity bonuses and inter-platform interference penalties that guide IoT devices to the platform where they are most likely to achieve high SINR and timely task completion. The platform-aware task distribution algorithm implements differentiated processing thresholds that reflect the asymmetric energy profiles of battery-limited UAVs and the grid-powered BS, maximising computational utilisation at the BS tier while protecting UAV battery reserves. Extensive Monte Carlo simulations validated the proposed framework against the two-tier UAV–HAP, demonstrating approximately $30\,\%$ higher total computed data volume, 15 percentage points higher task completion rate, and $20\,\%$ lower average end-to-end processing delay at peak load. The cumulative distribution function (CDF) analysis of received *SINR* confirmed an 8–12 dB improvement over unmanaged shared-spectrum deployment and maintained $85\,\%$ of devices above the 5 dB minimum decoding threshold, compared to only $30\,\%$ in the unmanaged scenario and $65\,\%$ in the two-tier baseline. The total system execution time of approximately 1.46 s per optimization cycle provides ample margin for operation at the required re-optimization frequencies.

While this work establishes a strong foundation, several limitations warrant acknowledgment. The present framework assumes fixed UAV hover positions chosen a priori based on hotspot geometry. Jointly optimizing three-dimensional UAV trajectories alongside the RIS phase configuration, stable matching, and task distribution, using, for example, successive convex approximation (SCA) or deep reinforcement learning (DRL), could further enhance hotspot coverage, reduce path loss, and improve the interference geometry for the sub-array suppression mechanism. This is particularly relevant for time-varying IoT spatial distributions, where adaptive positioning could yield substantial additional gains. The single-BS architecture studied here represents a first step toward full terrestrial integration. Extending the framework to multi-cell deployments with multiple BS-RIS panels, inter-cell interference coordination, and handover management would be a natural generalisation toward realistic 6G infrastructure scenarios. The three-way stable matching framework is readily extendable to multi-BS settings, and the sub-array suppression mechanism could be adapted to coordinate RIS panels across multiple base stations. The proposed framework exclusively employs passive RIS panels that shift phases without amplification. Hybrid active-passive RIS designs which embed low-power amplifiers in a subset of elements to compensate for the “multiplicative path-loss” effect, could extend the effective range of RIS-enhanced links, particularly for the UAV-to-BS and UAV-to-HAP backhaul segments operating over long distances. Regarding UAV battery replenishment, the proposed architecture achieves system-level persistent coverage primarily through the grid-powered BS tier, which operates continuously and independently of UAV energy state. For the hotspot sub-region, a staggered rotation schedule among the $|\mathscr {U}|=4$ UAVs ensures at least 75% of peak capacity is maintained during individual battery replenishment cycles. Formal optimization of UAV rotation schedules and the associated impact on hotspot coverage continuity is identified as a direction for future work.

## Data Availability

The datasets used and/or analyzed during the current study are available from the corresponding author on reasonable request.
